# A survey of the complex transcriptome from the highly polyploid sugarcane genome using full-length isoform sequencing and de novo assembly from short read sequencing

**DOI:** 10.1186/s12864-017-3757-8

**Published:** 2017-05-22

**Authors:** Nam V. Hoang, Agnelo Furtado, Patrick J. Mason, Annelie Marquardt, Lakshmi Kasirajan, Prathima P. Thirugnanasambandam, Frederik C. Botha, Robert J. Henry

**Affiliations:** 1 Queensland Alliance for Agriculture and Food Innovation, The University of Queensland, Room 2.245, Level 2, The John Hay Building, Queensland Biosciences Precinct [#80], 306 Carmody Road, St. Lucia, QLD 4072 Australia; 2 College of Agriculture and Forestry, Hue University, Hue, Vietnam; 3 Sugar Research Australia, Indooroopilly, QLD 4068 Australia; 4 ICAR - Sugarcane Breeding Institute, Coimbatore, Tamil Nadu India

**Keywords:** Sugarcane, Polyploid transcriptome, Transcriptome assembly, *De novo* assembly, Isoform sequencing, Hybrid assembly, SUGIT database

## Abstract

**Background:**

Despite the economic importance of sugarcane in sugar and bioenergy production, there is not yet a reference genome available. Most of the sugarcane transcriptomic studies have been based on *Saccharum officinarum* gene indices (SoGI), expressed sequence tags (ESTs) and *de novo* assembled transcript contigs from short-reads; hence knowledge of the sugarcane transcriptome is limited in relation to transcript length and number of transcript isoforms.

**Results:**

The sugarcane transcriptome was sequenced using PacBio isoform sequencing (Iso-Seq) of a pooled RNA sample derived from leaf, internode and root tissues, of different developmental stages, from 22 varieties, to explore the potential for capturing full-length transcript isoforms. A total of 107,598 unique transcript isoforms were obtained, representing about 71% of the total number of predicted sugarcane genes. The majority of this dataset (92%) matched the plant protein database, while just over 2% was novel transcripts, and over 2% was putative long non-coding RNAs. About 56% and 23% of total sequences were annotated against the gene ontology and KEGG pathway databases, respectively. Comparison with *de novo* contigs from Illumina RNA-Sequencing (RNA-Seq) of the internode samples from the same experiment and public databases showed that the Iso-Seq method recovered more full-length transcript isoforms, had a higher N50 and average length of largest 1,000 proteins; whereas a greater representation of the gene content and RNA diversity was captured in RNA-Seq. Only 62% of PacBio transcript isoforms matched 67% of *de novo* contigs, while the non-matched proportions were attributed to the inclusion of leaf/root tissues and the normalization in PacBio, and the representation of more gene content and RNA classes in the *de novo* assembly, respectively. About 69% of PacBio transcript isoforms and 41% of *de novo* contigs aligned with the sorghum genome, indicating the high conservation of orthologs in the genic regions of the two genomes.

**Conclusions:**

The transcriptome dataset should contribute to improved sugarcane gene models and sugarcane protein predictions; and will serve as a reference database for analysis of transcript expression in sugarcane.

**Electronic supplementary material:**

The online version of this article (doi:10.1186/s12864-017-3757-8) contains supplementary material, which is available to authorized users.

## Background

Understanding of the sugarcane transcriptome is limited due to the complexity in gene copy number, repetitive content, and heterozygosity in the genome [1, 2]. It is not clear how many transcript isoforms result from the alternative splicing in this potentially very complex transcriptome. Sugarcane is a polyploid hybrid between *Saccharum officinarum* and *S. spontaneum*, and each sugarcane hybrid has its own unique chromosome set (ranging from 80 to 130), containing up to 12 copies of each gene and a total ~35,000 predicted genes [3, 4]. Therefore, it is expected that sugarcane transcripts represent transcription of genes/homoelogues that are not only unique to the progenitor genomes but also transcription from alternate splicing, which we collectively refer to as transcript isoforms. Sugarcane genes such as those in the sucrose phosphate synthase (SPS) gene families [5, 6], invertase genes [7] and sucrose synthase family [8, 9] have been shown to be comprised of many isoforms. Most of the sugarcane studies, including transcriptome studies found in the literature, i.e. in [10] and [11], have been based on sorghum genomic/transcript sequences [12] which have the highest gene synteny and orthologous alignment with the sugarcane genome [13]; sugarcane expressed sequence tags (ESTs) [14]; *Saccharum officinarum* gene indices - SoGI v3.0 [15] representing ~90% of the estimated genes in *S. officinarum* [2, 3]; and other resources reviewed in [16, 17]. Studies based on these databases have provided useful information on the sugarcane transcriptome, while a whole genome sequence is not yet available. However, it is thought that there are still many sugarcane genes missing in these databases [18] and in addition, the full-length (FL) sequences of distinct transcript isoforms are not included. Use of these transcript databases for RNA-Seq analysis leading to the identification differentially expressed genes does not provide information on the corresponding isoform/s or the homoelogue/s contributing to the differential expression. There is a need to construct FL transcript sequences including such isoforms to facilitate analysis of isoform differential expression, and also to extend our understanding of the sugarcane transcriptome.

The transcriptome poses a great challenge when it comes to assembly and annotation. The differences in transcript abundance and the presence of different isoforms, greatly challenge the assembly of a transcriptome from short-reads (such as those from Illumina or Ion Torrent sequencing platforms); since the assemblers cannot distinguish between reads originally from different transcripts/isoforms carrying the same exons [19]. To date, most sequencing platforms offer a read-length which is shorter than the typical length of a eukaryotic mRNA (ranging between 1 and 2 kb, including a methylated cap at the 5’ end and poly-A at the 3’ end) [20]. The transcriptome sequences obtained from second generation sequencing technology (i.e. Illumina RNA-Sequencing, RNA-Seq) have been playing an important role in capturing the diversity in the RNA populations at a greater sequencing depth [10, 21, 22]. However, a precise prediction and identification of the alternative transcript splicing has not been possible. Algorithms in transcript splice-aware assemblers (i.e. Trinity [23], SOAP-denovo Trans [24], TransAbyss [25]) have been developed to detect splicing junctions and recover transcript isoforms by using information from short-reads, but these have not always been confirmed. That is, quite often these approaches overestimate and report spurious computational isoforms rather than picking up only biological ones. Overall, the assemblies from short-read data normally end up identifying more transcripts than expected (for an example, see [26]), which may be attributed not only to the diversity of RNAs and diversity of transcript/isoforms in the transcriptome, but also to the limitation in recovering FL transcripts. Most studies use these tools, then filter the transcripts through clustering by retaining the longest sequence in each cluster as representative for analysis, and consider them to be the major isoforms or unigenes [10, 21]. Current algorithms such as in Bernard et al. (2014) for isoform identification and quantification require longer reads and cannot tackle genes with too many exons. With the advent of third generation sequencing technology, the cost-per-transcriptome has been reduced, whereas the length of the sequencing reads has been increased significantly. As of August, 2016, the average read length of PacBio Single Molecule, Real-Time (SMRT) sequencing is >10 kb and real length can be up to 60 kb (PacBio, Menlo Park, CA, USA [27]. This technology provides an ability to generate long read transcripts and characterize them using the protocol called Isoform Sequencing (hereafter referred to as Iso-Seq). This protocol has been applied in some recent studies, for example, detecting 10,053 alternative splicing events in 27,860 unique transcripts (40.7% novel), covering ~89% of the total sorghum annotated genes [28]; and producing 111,151 unique transcripts (57% novel transcripts) in the maize transcriptome derived from six different tissues, covering ~70% of the annotated genes [29].

This study represents the first full-length transcriptome reference sequences from sugarcane derived from three different tissues, of different developmental stages, by using the PacBio long-read Iso-Seq technique. In addition, RNA-Seq was used to improve the PacBio transcript isoforms by short-read error correction, and comparison between sugarcane transcripts obtained/assembled from these two different platforms. Annotation of the sugarcane FL transcript isoforms could improve sugarcane genome models, contribute towards understanding of the complexity of the sugarcane genome and serve as reference sequences for differential expression analysis in the future.

## Results

### Sugarcane transcriptome from PacBio isoform sequencing

A pooled sample representing polyA RNAs from three tissues (leaf, internode and root), of different developmental stages (immature and mature) was sequenced to obtain a wide coverage of the sugarcane transcriptome. A total of 290,393 reads of inserts (ROIs) was generated, with a total of 548,763,750 nucleotides from six SMRT cells of non-normalized bins (0.5-2.5 kb, 2–3.5 kb, 3–6 kb and 5–10 kb) and normalized bins (0.5-2.5 kb and 2–3.5 kb), including 186,999 (64%) FL non-chimeric ROIs and 103,394 (36%) non-FL ROIs. The length of ROIs ranged from 300 bp to 53,235 kb, with an N50 of 2,408 bp. The length distribution of all ROI data is presented in Additional file 1: Figure S1. A total of 65,715 high quality sequences and 41,891 low quality sequences were obtained from Quiver polishing, referred to as polished transcript isoforms. The total unique, non-redundant transcript isoforms included 107,604 sequences, with the length ranging from 301 bp to 18,548 bp, N50 of 1,994 bp and N75 of 1,271 bp and 48.90% GC content.

### Improving PacBio transcript quality by error correction using RNA-Seq reads

We followed two error correction pipelines (proovread and LoRDEC), using three datasets from RNA-Seq derived from the same experiment; 586,360,045 non-normalized reads, 378,337,000 reads BBnorm-normalized and 213,165,230 trinity-normalized reads. Overall, the error correction led to improvement in transcript prediction, more transcripts covered the full-length of known proteins, longer open reading frames (ORFs), better completeness results in CEGMA/BUSCO assessments, and higher alignment rate of transcript isoforms to the sorghum genome. This only resulted in a slight change in the total number of transcripts isoforms after removing all exact 100% identical sequences. The LoRDEC error correction outperformed proovread with our transcript data. The best corrected set was derived from LoRDEC using the Trinity-normalized reads, which resulted in 42.9% of the total transcripts with ORFs passing the Evigene score in transcript prediction, while that of the non-corrected transcripts was only 14.6% (Table 1, for details, see Additional file 1: Table S1). There were 252,491 ORFs (with a N50 of 888 bp) detected by TransDecoder in this corrected PacBio transcript isoform set, compared to 243,637 ORFs (N50: 570 bp) for the non-corrected dataset. The retained ORFs with Pfam and *Viridiplantae* protein hits from the corrected dataset had an N50 of 1,158 bp, while that of the non-corrected was 708 bp. The CEGMA and BUSCO alignments showed that the corrected PacBio dataset had a higher completeness level than the non-corrected (CEGMA: 98% and 96%; BUSCO: 90% and 87%, respectively). About 69.4% of the corrected transcripts aligned to the sorghum genome, while the alignment of the non-corrected transcript isoforms was 66.4%. We chose the PacBio dataset corrected by LoRDEC, using reads normalized by Trinity (hereafter referred to as PacBio transcript isoforms), for downstream analysis. This final PacBio transcript isoforms set had 107,598 sequences after removal of strictly identical sequences; of total length ~193 Mb, with individual transcript length ranging from 300 to 18,302 bp, N50 of 1,991, N75 of 1,269, and 49.02% GC content.

**Table 1:** Summary of correction of PacBio transcript isoform data using Illumina short-reads

Analysis		PacBio non-corrected	LoRDEC Trinity normalized reads
Total transcripts		107,604	107,598
Evigene prediction	Okay transcripts	18,190	51,025
	Main transcripts	14,124	25,012
	Alternate transcripts	4,066	26,013
	GC%	61.8	51.4
CEGMA alignment (%)		96.4	97.98
BUSCO notation (%)		87.13	90.27
ORFs detected	Minimum 300 bp	243,637	252,491
	ORF N50 (bp)	570	888
Protein counts covered ≥90%		9.727	12,611
Transcripts mapped to sorghum genome (%)		66.43	69.44

### *De novo* assembly of the sugarcane transcriptome from short-reads

The assembly of the sugarcane transcriptome from Illumina RNA-Seq short-reads was carried out to provide a comparative reference for the transcript isoform sequences obtained from PacBio Iso-Seq, since RNA-Seq has been utilized widely in construction of transcriptomes. Of 1,500 million total raw reads generated (paired distance estimated range of 64–302 bp), 1,015,845,414 reads survived after trimming, having a quality score cutoff of Q30. Trinity normalization retained only 6% (59,054,880 reads) at a maximum coverage of 50, while retaining 15% (150,412,240 reads) and 21% (213,165,230 reads) at maximum coverage of 400 and 2,000, respectively. A QC report of all read datasets was generated by FastQC v0.11.5 [30] (Additional file 1: Figure S2). The use of BBnorm was selected due to the fact that Trinity normalization heavily reduced the reads compared to BBnorm at the same maximum coverage cutoff. About 37% (378,337,000 reads) of the total reads remained at maximum coverage 10,000 by BBnorm package for *de novo* assembly.

After assembly and individual clustering, four initial assemblies were obtained from Trinity, CLC-GWB, Velvet/OASES, and SOAPdenovo-Trans, respectively. We observed varied contig number, N50, cumulative length and length distribution in each of the assemblies. The total number of contigs from the Trinity-assembly was 431,255 (N50: 2,194 bp), while that from the CLC-GWB assembly was 508,239 (N50: 1,014 bp), Velvet/Oases assembly gave 798,345 (N50: 516 bp) and SOAPTrans assembly 289,705 (N50: 674 bp). Table 2 summarizes the *de novo* assembly results in this study, including all major statistics and QC, more details see Additional file 1: Figure S3. The final total number of contigs clustered by CD-HIT-EST at 95% identity and after retaining transcripts with length from 300 bp to 10 kb, was 906,566, of ~967 Mb, having an N50 of 1,671 bp, N75 of 745 bp and 43.67% GC. This clustered assembly was referred to as the *de novo* transcript contigs.

**Table 2:** Comparison of *de novo* assemblies used in this study

Assembly	Trinity	CLC-GWB	Velvet/OASES	SOAPdenovo-Trans	Final assembly
# contigs (> = 0 bp)	431,255	508,239	798,345	289,705	906,566
# contigs (> = 1000 bp)	210,220	109,992	37,698	34,781	294,867
# contigs (> = 2000 bp)	104,013	34,970	2,633	4,817	130,095
# contigs (> = 3000 bp)	46,942	13,732	441	1,099	57,437
# contigs (> = 4000 bp)	19,218	5,574	94	282	23,416
# contigs (> = 5000 bp)	7,542	2,413	31	104	9,227
# contigs	431,255	508,239	798,345	289,705	906,566
Largest contig	28,461	29,928	11,272	18,497	9,990
Total length	608,060,518	419,587,279	409,817,309	182,675,172	966,867,516
GC (%)	43.88	43.50	42.84	43.25	43.67
N50	2,194	1,014	516	674	1,671
N75	1,216	542	389	455	745
L50	89,618	107,715	266,636	82,953	168,723
L75	181,071	253,430	497,065	165,967	385,929
# N’s per 100 kbp	0	0	0	0	0

**Fig. 1:**
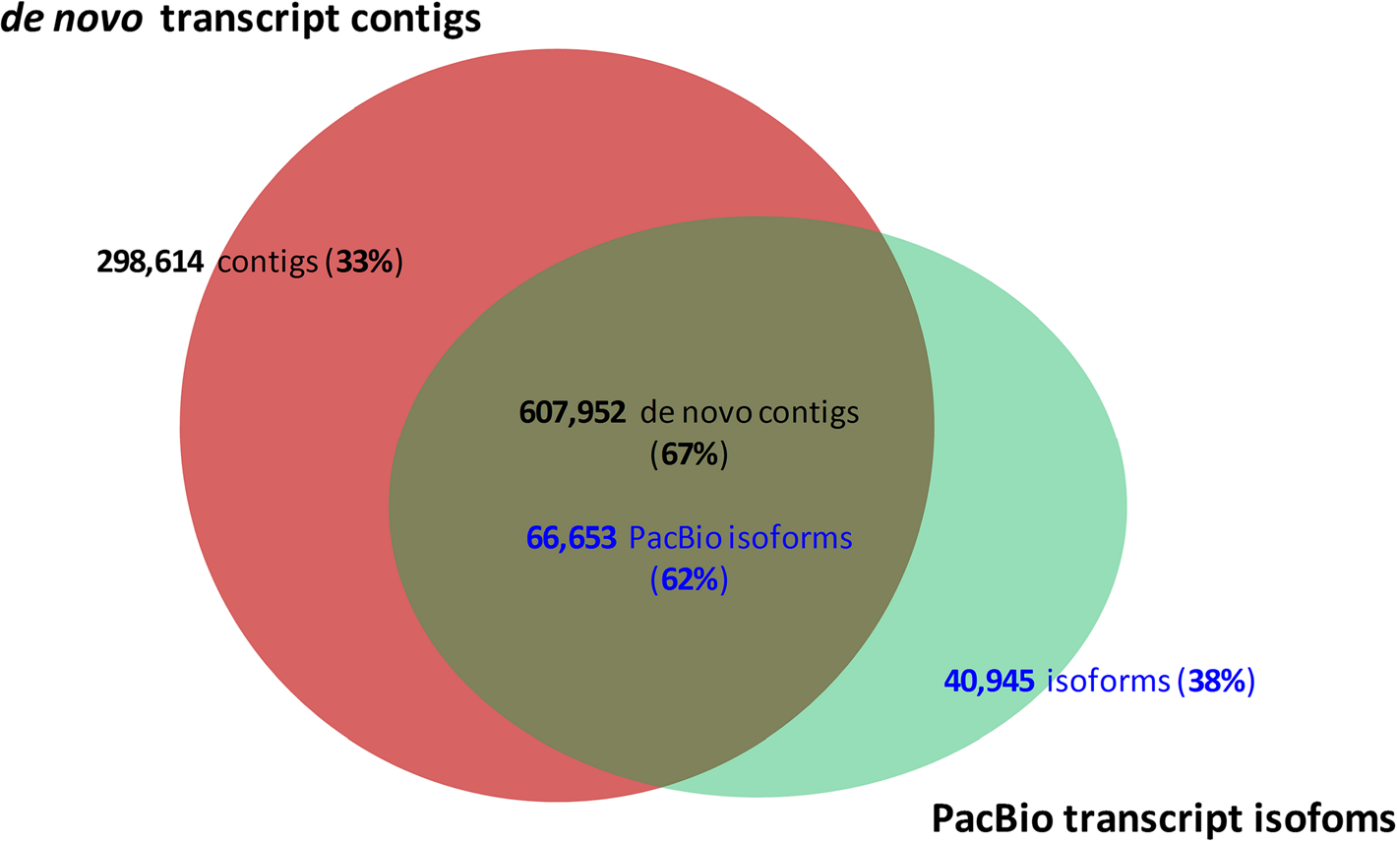
Comparison between the sugarcane PacBio transcript isoforms and *de novo* transcript contigs

When compared to PacBio transcript isoforms (Fig. 1) by BLASTN (e-value ≤1e-20, pairwise identity ≥75%, min bit score ≥100), 67.1% of the *de novo* transcript contigs (607,952 contigs) exhibited similarity to 61.9% of the PacBio transcript isoforms (66,653 isoforms). There were 32.9% of *de novo* transcript contigs (298,614) and 38.1% of PacBio transcript isoforms (40,945 isoforms) that were unique to each of the datasets.

### Analysis of reads mapping back to transcripts

**Fig. 2:**
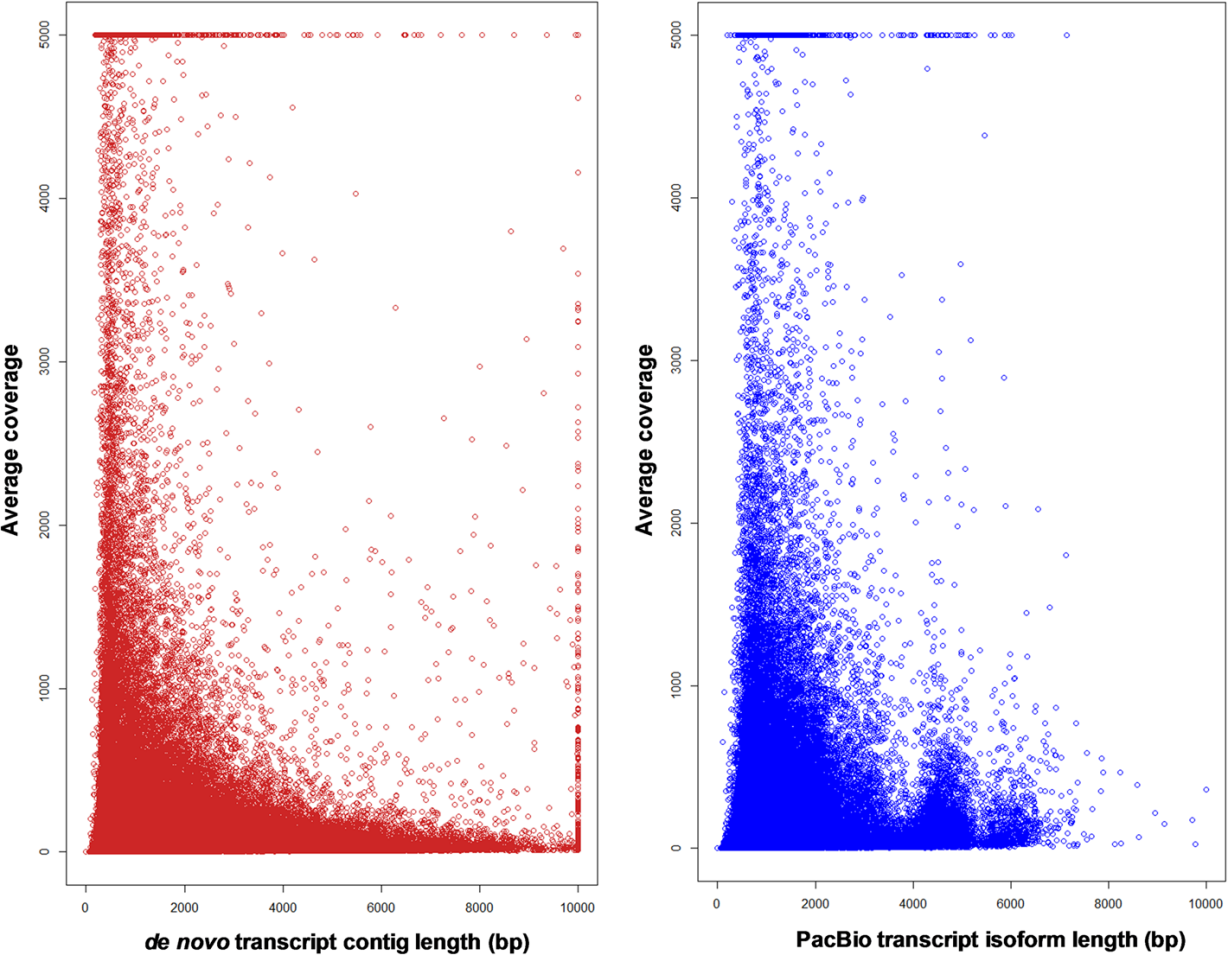
Average coverage of sugarcane *de novo* contigs and PacBio isoforms obtained from read mapping. **a**, Coverage of *de novo* transcript contigs. **b**, Coverage of PacBio transcript isoforms

In mapping back the RNA-Seq read data to transcripts from both PacBio and *de novo* transcripts, we observed 86.8% of reads mapped back to PacBio transcript isoforms, while 98.5% mapped to the *de novo* transcript contigs. Figure 2 shows the average coverage plotted against the transcript length for both assemblies.

### Transcriptome completeness analysis

In both CEGMA and BUSCO alignments (Table 3), the PacBio dataset showed a lower completeness level than the *de novo* dataset. The PacBio transcripts had 96.8% CEGMA alignment (98.0% including partial CEGMA alignment), and the *de novo* assembly had 97.6% CEGMA alignment (100% including partial CEGMAs). There were no missing CEGMA in the *de novo* transcript contigs, and there was 2.0% missing CEGMAs (five out of 248 CEGMA proteins) in the PacBio dataset. Similarly, in BUSCO alignment to 956 conserved proteins, the PacBio transcript isoforms had lower completeness than that of *de novo* assembly, by having 83.6% completeness compared to 93.0% (90.3% and 97.7%, respectively, when fragmented BUSCOs were counted). The *de novo* assembly had a higher level of duplication in the BUSCO alignment, suggesting that the assembly contains duplicate contigs of different lengths (defined as isoforms) assembled by different assemblers and retained after clustering. Using the same CEGMA and BUSCO protein alignments, we assessed the unigene dataset from [10] and the SoGI database to determine the consistency of the methods. The unigenes had 90.3% (95.6% including partials) CEGMA completeness, 79.6% (91% including fragmented) BUSCO completeness; and the SoGI dataset had 62.9% (87.5%) CEGMA completeness and 46.7% (81.1%) BUSCO alignment completeness. The SoGI dataset had the largest proportion of partial/fragmented (24.6% CEGMA and 34.4% BUSCO) and missing proteins (12.5% CEGMA, 18.93% BUSCO), since this dataset contained gene indices and ESTs (fragmented mRNAs).

**Table 3:** Transcriptome coverage analysis based upon CEGMA and BUSCO alignment

CEGMA alignment										
Assembly	Count	# CEGs Protein	Complete CEGs count	% Completeness	Partial CEGS	% Partials	Missing CEGs	% Missing	Total CEGs	% Complete and partial CEGs
PacBio isoforms	107,598	248	240	**96.77**	3	1.2	5	2.02	243	**97.98**
*de novo* contigs	906,566	248	242	**97.58**	6	2.4	0	0.00	248	**100.00**
Unigenes	72,269	248	224	**90.32**	13	5.2	11	4.44	237	**95.56**
SoGI dataset	121,342	248	156	**62.90**	61	24.6	31	12.50	217	**87.50**
BUSCO notation alignment										
Assembly	Total complete (%)	Single copy BUSCOs (%)	Duplicated BUSCOs (%)		Fragmented BUSCOs (%)		Missing BUSCOs (%)		Complete and fragmented (%)	
PacBio transcript isoforms	**83.58**	17.15	66.42		6.69		9.73		**90.27**	
*de novo* transcript contigs	**92.99**	10.04	82.95		4.71		2.30		**97.70**	
Unigenes	**79.60**	63.81	15.79		11.40		9.00		**91.00**	
SoGI	**46.65**	26.67	19.98		34.41		18.93		**81.07**	

### Counting the full-length and nearly full-length transcripts

**Fig. 3:**
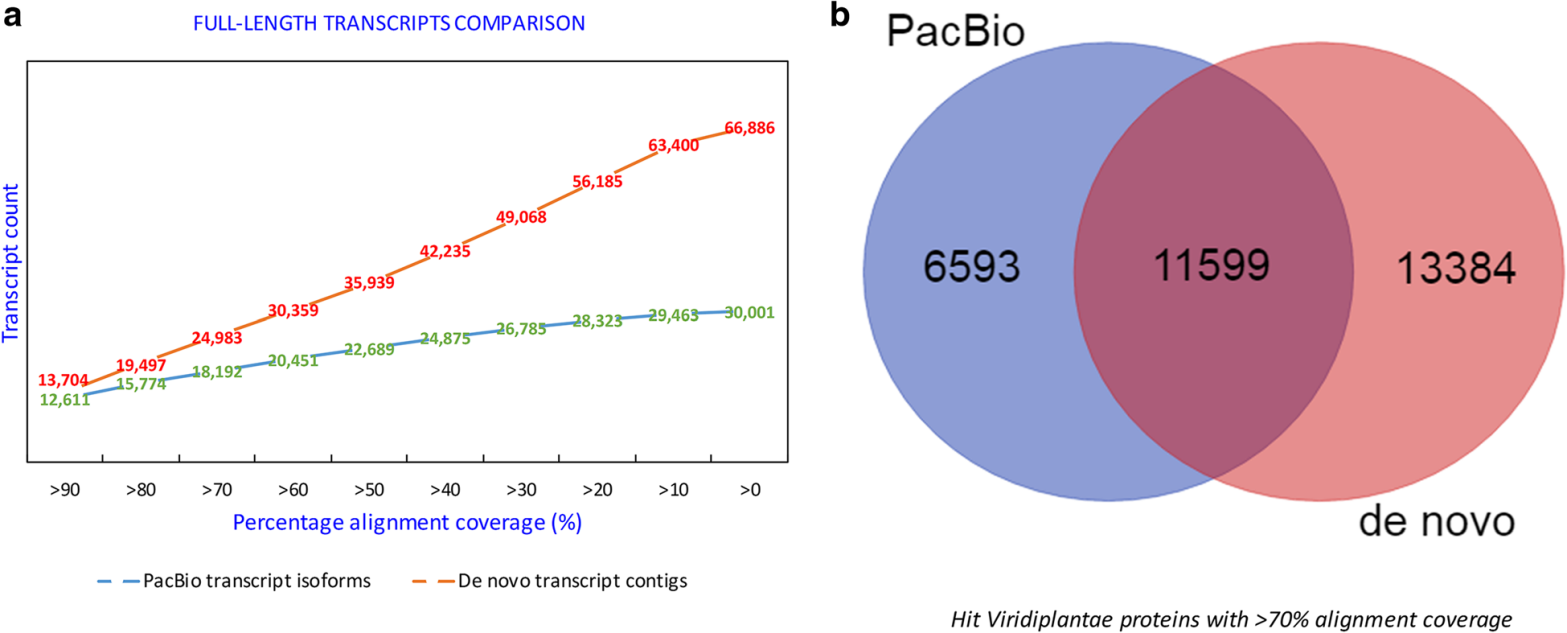
Full-length analysis between sugarcane PacBio transcript isoforms and *de novo* transcript contigs. **a**, Counts of proteins covered by transcripts at different thresholds. **b**, Comparison between the protein hits from PacBio and *de novo* transcripts which covered at least 70% *Viridiplantae* protein length

The number of transcripts appearing to be full-length with at least 90% and 70% alignment coverage of the *Viridiplantae* UniProt proteins was estimated and compared between PacBio transcript isoforms and *de novo* transcript contigs (Fig. 3). The PacBio dataset had 12,611 transcripts appearing to be full-length (≥90% alignment coverage), and 18,192 transcripts (≥70% alignment coverage of the known proteins), and that of the *de novo* transcript contigs were 13,704 and 24,983 at 90% and 70%, respectively. Analysis of the matched proteins at the cutoff of 70% alignment coverage from both assemblies indicated that only 11,599 proteins (37% total hits) were common between these two assemblies, with 6,593 (21%) unique to PacBio dataset, and 13,384 (42%) unique to the *de novo* transcript contigs.

As these results considered each protein from the UniProt database as only one count, regardless of the presence of different isoforms that carry the same protein sequence part (i.e. represented in the database as only domain part) getting matched many times. A modified approach to counting the full-length transcripts for isoforms was applied, in which we took all counts of isoforms that hit the same protein into consideration and estimated the number of full-length proteins covered. Using this strategy, we found in the PacBio data, 39,999 transcripts that covered ≥90% of *Viridiplantae* proteins and 59,725 transcripts that covered ≥70% of *Viridiplantae* proteins. In the *de novo* transcript contigs, it was 33,762 and 76,865 protein hits covered by ≥90% and ≥70%, respectively. *De novo* transcript contigs had more proteins covered at lower percentage due to the greater duplication retained in the assembly, and inclusion of more partial gene content. When protein hits of ≥90% alignment coverage from the two results were compared, the unique protein hits of PacBio was 12,611 and *de novo* was 13,704, which were the same as in the first approach.

Investigation of 164 full-length genes from sugarcane and other grass species showed that PacBio dataset resulted in a better performance in terms of recovering the full-length sequence of these specific genes (Table 4). At an e-value ≤1e-20, there were more genes covered by transcripts at 90% (103 genes) and 70% (144 genes) in PacBio transcript isoforms than that in *de novo* transcript contigs (87 genes and 130 genes), unigene set (48 and 89 genes) and SoGI database (22 and 55 genes), respectively. The lower full-length gene count in unigenes could be due to only main isoforms being retained for this dataset, while lower full-length gene count in SoGI database could be due to the fraction of ESTs in it.

**Table 4:** Alignment and full-length assessment of a selected gene set

Gene alignment covered (%)	Transcripts counts			
	PacBio transcript isoforms	*De novo* transcript contigs	Unigenes	SoGI
>90	103	87	48	22
>80	133	118	63	37
>70	144	130	89	55
>60	151	137	111	73
>50	158	147	122	100
>40	162	150	137	123
>30	163	153	144	142
>20	163	161	150	156
>10	164	162	155	164

### Evidence of alternative splicing in the sugarcane transcriptome from PacBio Iso-Seq

**Fig. 4:**
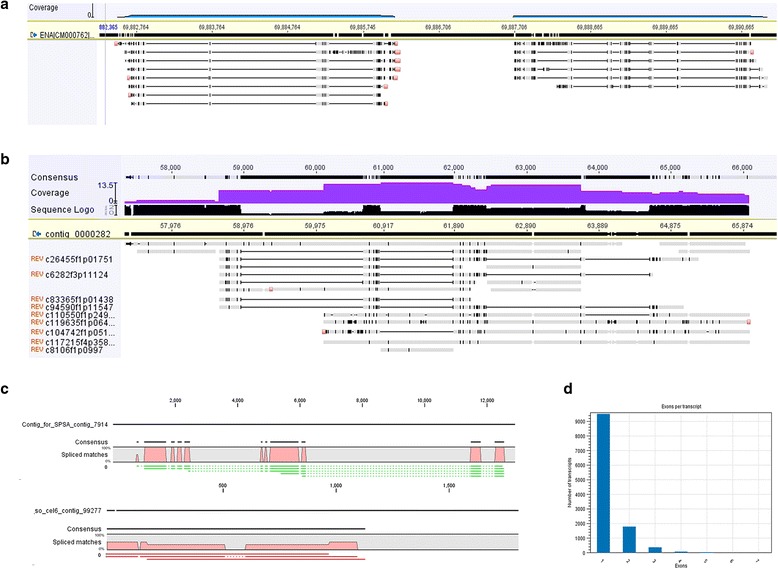
Evidence of different transcript isoforms of sugarcane transcriptome present in the PacBio transcript dataset. **a**, Isoforms aligned against the sorghum chromosome 1. **b**, Isoforms aligned to contigs of our in-house sugarcane whole genome *de novo* assembly. **c**, Different transcript isoforms aligned to sucrose phosphate synthase gene and cellulase 6 gene contigs. **d**, Average exons per transcript estimated based on the transcript isoforms aligned against sorghum genome

Using the results from PacBio transcript isoforms mapped against the sorghum genome (Fig. 4a), our in-house sugarcane whole genome assembly from sugarcane cultivar Q155 (Fig. 4b) and particular contigs that spanned through the sucrose phosphate synthase A and cellulase 6 genes (Fig. 4c), we were able to visualize alternative splicing in the PacBio transcript isoforms. Using the Transcriptome Analysis Pipeline for Isoform Sequencing (TAPIS) described in [28], we detected amongst those transcript isoforms aligned against the sorghum genome, 4,870 alternative splicing events, including 1,302 (26.7%) intron retention, 559 (11.5%) skipped exon, 1,365 (28.0%) alternative 5’splice sites and 1,644 (33.8%) alternative 3’splice sites. An estimation of exons per transcripts amongst the transcript isoforms aligned against the sorghum genome is also included (Fig. 4d).

### Prediction of potential coding regions, main and alternate transcripts analysis

We analyzed the candidate coding regions in the transcript sequences by retaining only open reading frames (ORFs ≥100 aa) that exhibited homology with the Pfam protein domain database or the UniProt *Viridiplantae* known protein database, which were more likely to be biologically real. There were 252,491 non-overlapped ORFs detected in the PacBio transcript isoform sequences, belonged to 100,639 ORF-containing transcript isoforms (93.5% of the total). Only 6,959 isoforms did not contain ORFs and these were used for characterization of non-coding RNAs, in the next Section. Of the total ORF-containing transcripts, 92,448 matched the *Viridiplantae* proteins (e-value ≤1e-5), while 87,168 matched the Pfam protein domains. The total number of transcript sequences retained in combination with the TransDecoder frame-score was 96,114 (89% of total transcript sequences), with lengths ranging from 300 bp to 8,142 bp, and N50 of 1,158 bp. There were more sequences retained in the *de novo* assembly, since it started with more data, however transcript contig length was shorter than that of the PacBio dataset. A total 747,912 ORFs were found in 491,544 ORF-containing contigs (54.2% of the total *de novo* assembly). When combined with the homology search results, 279,623 transcript contigs matched the *Viridiplantae* protein database, and 232,567 contigs matched the Pfam protein domains. The final number of contigs retained by TransDecoder was 355,453, accounting for 39.2% of the total *de novo* assembly. This final contig set had lengths ranging from 300 bp to 9,501 bp and N50 of 738 bp.

**Fig. 5:**
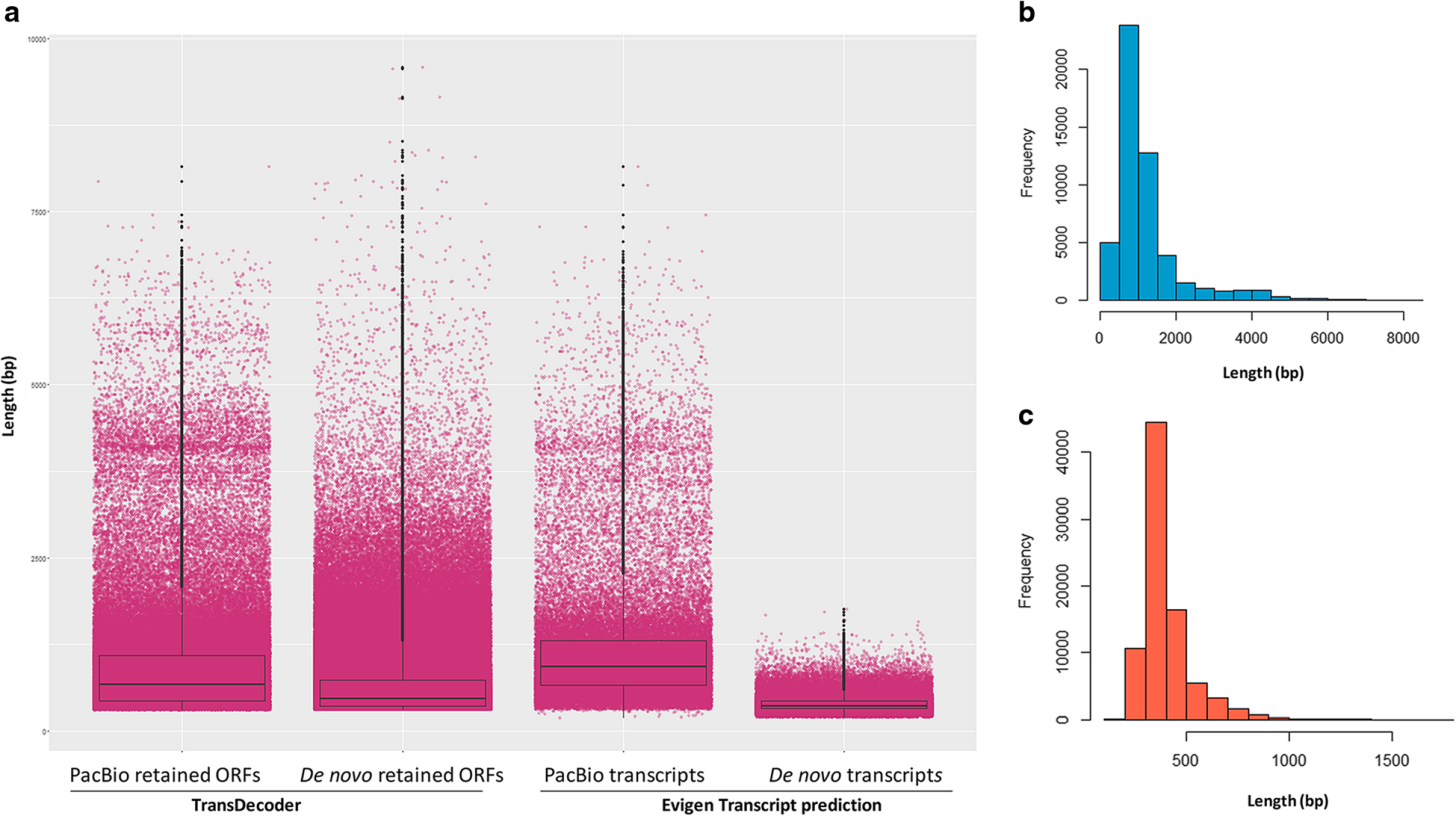
Analysis of ORFs and transcript prediction of sugarcane transcriptome. **a**, Length distribution of ORF-containing transcripts resulted from TransDecoder and Evigen. **b**, Length distribution of predicted transcripts by Evigene in PacBio data. **c**, Length distribution of predicted transcripts by Evigene in *de novo* contig data

When predicting the potential coding genes using the Evigene pipeline, the total number of predicted transcripts in the PacBio transcript isoforms was 51,025 (from 43% of ORFs detected by the program, including, 25,012 categorized as main transcripts, and 26,013 as alternate transcripts), while the dropset had 67,730 transcripts (57%). The average length of the largest 1,000 proteins from the dataset was reported to be 1,348 aa, and all candidate transcripts (main and alternate) had an N50 of 1,296 bp and CDS length ranged from 186 bp to 8,142 bp. As for the prediction by TransDecoder, the *de novo* contig set had more ORFs, and therefore, more predicted transcripts both main and alternate. There were 83,041 predicted transcripts (10.5% of total ORFs, including 56,766 main and 26,275 alternate transcripts) with an N50 of 384. Compared to PacBio transcript isoforms, the *de novo* predicted transcripts had much shorter length distribution and average length of the largest 1,000 proteins (298 aa). Using the same prediction approach on the unigenes and SoGI, we found 13,205 predicted main transcripts in the unigenes (without alternative forms, since this only contained the major isoforms reported by Trinity), and 41,042 predicted transcripts (32,013 main and 9,029 alternate transcripts) in SoGI. The average length of the largest 1,000 proteins for the unigenes was 298 aa, while that of SoGI was 287 aa. All the results of ORF and transcript prediction are presented in Table 5 and Fig. 5.

**Table 5:** Open reading frame and transcript prediction analysis of sugarcane transcriptome sequence data

ORF prediction	PacBio transcript isoforms	*De nov*o transcript contigs		
ORF containing transcripts	100,639	491,544		
Retained transcripts^a^	96,114	355,453		
Min length	300	300		
Max length	8,142	9,501		
N50	1,158	738		
Evigene prediction	PacBio transcript isoforms	*De novo* transcript contigs	Unigenes	SoGI
Total transcripts	51,025	83,041	13,205	41,042
Main transcripts	25,012	56,766	13,205	32,013
Alternate transcripts	26,013	26,275	0	9,029
Ave length 1 K proteins^b^	1,348	298	298	287

^a^transcripts with Pfam and *Viridiplantae* hits. ^b^Average length (aa) of the largest 1.000 proteins

### Analysis of candidate non-coding RNAs

The proportion of candidate non-coding transcripts (with a length ≥300 bp but containing no detected ORF ≥100 aa) was different between the PacBio dataset (6.5%, 6,959 sequences) and *de novo* dataset (45.8%, 415,022 sequences). Due to a large number of non-coding contigs in the *de novo* contig dataset, which were likely from different non-coding RNA classes, such as transfer RNAs (tRNAs), small RNAs, microRNAs (miRNAs) and ribosomal RNAs (rRNAs), and also *de novo* assembly artifacts; only the candidate long non-coding transcripts from the PacBio (which were attributed to polyA non-coding RNAs) were used for further characterization. The PacBio non-coding transcript set had a length ranging from 300 to 7,336 bp. When compared against the NCBI nucleotide NR database, it was found that 174 transcripts matched sequences from bacterial, fungal and insect sources. The remaining 6,785 sequences included 5,565 sequences matching the NCBI NR nucleotide database, and 1,220 transcript isoforms that did not match any entries in NR database. A total of 4,276 sequences exhibited similarity to protein-encoding sequences (these were likely results of sequencing errors that disrupted the code and prevented the detection of an ORF ≥100 aa), and 1,206 sequences matched NCBI non-coding entries belonging to genomic sequences of the grass family. These 1,206 sequences contained 96 transcript sequences matching the predicted non-coding RNAs of *Zea mays* and *Setaria italica*. The final retained candidate long non-coding RNAs for this dataset were 2,426 transcript sequences (2.3%), including 1,220 non-ORF, non-blast hit transcript sequences (Additional file 1: Figure S4).

### Repeat content analysis

The repeat masking against the customized repeat library for *Viridiplantae* showed that the total number of interspersed repeats within the PacBio transcript isoform data was 30,243 (accounting for 4.8% of total bases); including 50.2% retroelements, 41.1% DNA transposons and 8.7% unclassified repeat elements. The retroelements included short interspersed nuclear elements - SINEs (1.5%), long interspersed nuclear elements - LINEs (13.8%) and long terminal repeat (LTR) elements (34.8%). Amongst all repeat classes, the LTR Gypsy/DIRS1 was the most abundant which made up to 17.9%, following by LTR Ty1/Copia (12.6%), LINEs L1/CIN4 (10.4%) and DNA transposon Tourist/Harbinger (9.7%). In the *de novo* dataset, there were 317,305 interspersed repeats identified (8.0% of total bases), including 46.0% retroelements (13.1% SINEs, 30.7% LTR elements), 48.9% DNA transposons and 5.1% unclassified repeats). Gypsy/DIRS1 (17.4%), Tourist/Harbinger (15.1%), Ty1/Copia (12.5%) and LINEs L1/CIN4 (8.98%) were the dominant repeats in the *de novo* dataset. All details are presented in Additional file 1: Table S2.

A total of 15,715 SSRs were discovered in 13,356 PacBio transcript isoforms (12.4%), while a total of 52,847 SSRs were identified in 48,091 *de novo* contigs (5.3%). In both cases, the most abundant motifs detected were tri-nucleotide (66.4%, and 52.7% of the total SSRs, respectively), followed by di-nucleotide motifs (27.0% in PacBio dataset and 41.1% in the *de novo* dataset). The SSRs detected in both PacBio transcript isoforms and *de novo* transcript contigs are presented in Additional file 1: Table S3.

### Transcript annotation

Using 104,998 PacBio transcript isoforms (97.6%) (after filtering out 2,426 candidate non-coding RNAs and 174 sequence contaminants from microbes identified in the previous Section), we found that there were 528 additional sequences from other sources, such as bacteria, fungi and insects, present in the samples. This made up a total of 702 cross-contaminated sequences (0.6%) in the original dataset and these were subsequently removed prior to functional annotation. Of 104,470 remaining sequences, 102,020 (94.8%) matched the NCBI NR nucleotide database, and 2,450 sequences that did not return any matches while containing an ORF, which could potentially be novel transcripts in the sugarcane transcriptome. When compared against the *Viridiplantae* protein database, 99,313 transcript isoforms (92.3%) showed similarity against 30,001 plant protein sequences. There were 97,997 transcript isoforms (91.1%) of PacBio transcript isoforms matching 19,057 sorghum proteins, 96,523 (89.7%) matching 22,231 SUCEST entries (when filtered for pairwise similarity of 75%, min score of 100 and an e-value <1e-20, 88,694 (82.4%) transcript isoforms remained). The comparison between PacBio transcript isoforms and *de novo* transcript contigs in Table 6, showed that *de novo* contig dataset matched more *Viridiplantae* proteins (67,162), sorghum proteins (28,901) and SUCEST entries (36,501 sequences).

**Table 6:** Annotation of sugarcane transcriptome

Assembly		Database		
		*Viridiplantae* proteins^a^	Sorghum proteins^a^	Sugarcane EST (SUCEST)^a^
PacBio transcript isoforms	Transcript isoforms matched	99,313	97,997	96,523
	% transcript isoforms	92.30	91.08	89.71
	Number of proteins matched	30,001	19,057	22,231
	% proteins in database		40.37	47.61
*De novo* transcript contigs	Transcript contigs matched	314,814	276,423	546,177
	% transcript contigs	34.73	30.49	60.25
	Number of proteins matched	67,162	28,901	36,501
	% proteins in database		61.22	84.59

^a^at an e-value ≤1e-5

There were 504 PacBio transcript isoforms matching the sugarcane chloroplast genome, and 542 matching the sorghum and maize mitochondrial genomes, while of the *de novo* transcript contigs, 377 matched the chloroplast genome and 658 matched the mitochondrial genome (only hits with an e-value = 0.0 were considered). Even though chloroplast and mitochondrial reads were removed prior to *de novo* assembly to reduce the read abundancy, there could be some chloroplast and mitochondrial reads still remaining in the RNA-Seq data for which a stringent setting and the mitochondrial genomes from closely related species were used for mapping. Using the plant transcription factor (TF) database, we identified a total of 1,669 TFs in the PacBio transcript isoforms, including 1,006 similar to those in sorghum, 503 similar to maize TFs, 130 similar to rice TFs and 30 similar to TFs in other plant species (Table 7). There were 664 additional TFs identified using the Grassius sugarcane TFs, and all 2,333 identified TFs were distributed on 7,886 TF-encoding transcript isoforms, which accounted for ~7.3% of the total sequences. These TFs were from 80 annotated TF families, and their distribution is presented in the Additional file 1: Figure S5. In the *de novo* transcript contigs, we identified 4,177 TFs from 78 TF families, belonging to 33,268 TF-encoding transcript contigs. Two families (SRS and S1Fa-like) were not found in the *de novo* transcript contigs, compared to the PacBio dataset.

**Table 7:** Sugarcane transcription factors analysis of PacBio transcript isoforms

Species	Count
Sorghum	1,006
Sugarcane - Grassius	664
Maize	503
Rice	130
Others	30
Total transcription factors	2,333
Total families	80
Total TF-encoding transcript isoforms	7,886

**Fig. 6:**
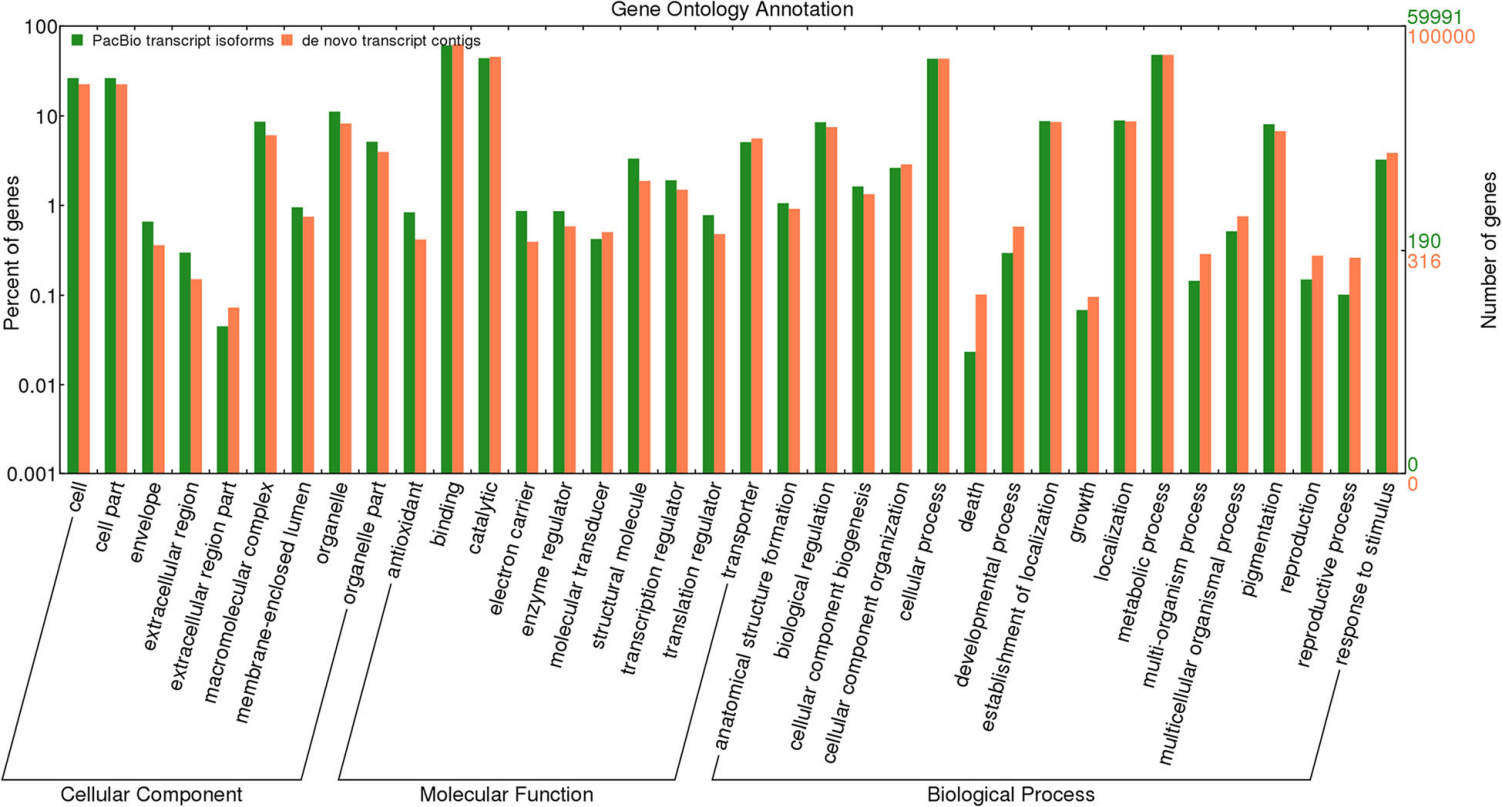
Gene ontology enrichment analysis of sugarcane transcript sequences. For *de novo* transcript contigs, only GO terms represented for 100,000 sequences were used

Using all ORF-containing transcripts in functional annotation, a total of 1,986 GO terms were assigned to 59,991 PacBio transcript isoforms (55.8% of total set). These GO terms were classified into three main classes, cellular component, molecular function and biological process. Among the cellular component category, the highest proportion of transcript isoforms was involved in cell and cell part (26.2%), organelle (11.2%) and macromolecular complex (8.6%). In molecular function, binding was dominant (60.7%), followed by catalytic activity (43.8%), transporter (5.1%), structural molecule activity (3.3%) and transcription regulator activity (1.9%). In biological process, the most transcript isoforms were assigned to metabolic process (47.7%), cellular process (43.1%), localization (8.9%), biological regulation (8.5%), pigmentation (8.1%), response to stimulus (3.3%) and cellular component organization (2.6%). A comparison of enriched GO terms between the PacBio transcript isoforms and *de novo* transcript contigs (which had 137,469 sequences annotated against 2,456 GO terms, accounting for 28% of total ORF containing *de novo* contigs and 15% of total *de novo* set) is presented in Fig. 6.

**Fig. 7:**
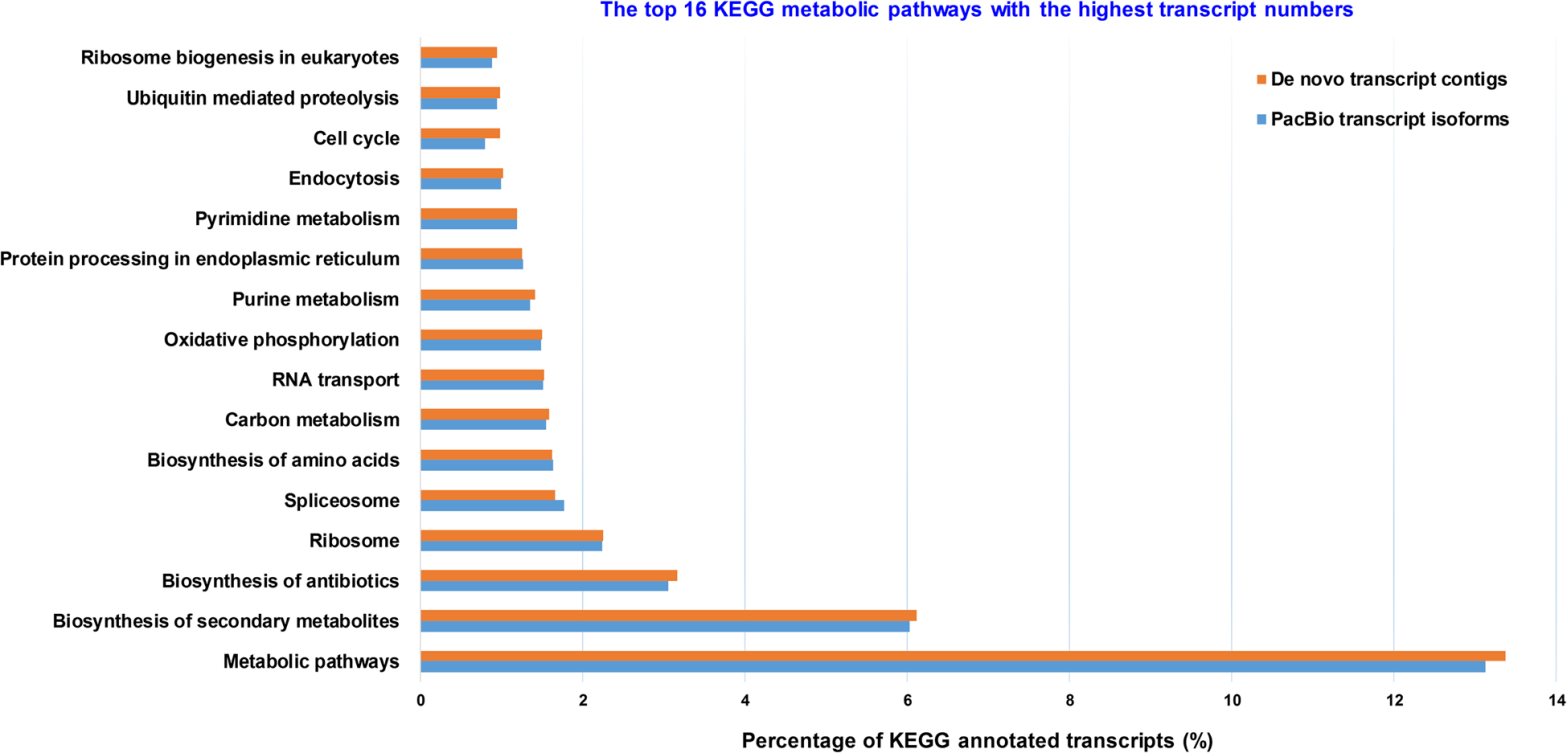
KEGG metabolic pathway classification of sugarcane PacBio transcript isoforms and *de novo* transcript contigs

KEGG metabolic pathway analysis provided additional possible functional information showing the pathways that the transcript isoforms take part in, since one gene could be assigned to more than one GO term in the Gene Ontology annotation. The results are presented in Fig. 7, expressing the percentage of transcripts involved in the pathways. A total of 24,334 PacBio transcript isoforms (~22.6% of the total) matched 3,233 KEGG pathway annotations (KOs), while 29,913 *de novo* transcript contigs (~3.3% of the total) matched 3,413 KO annotations. The largest functional pathway was metabolic pathway, representing 13.1% and 13.4% for PacBio and *de novo* transcript datasets, respectively; followed by biosynthesis of secondary metabolites (6.0%/6.1%), biosynthesis of antibiotics (3.1%/3.2%), ribosome (2.2%/2.3%), splicesome (1.8%/1.7%), biosynthesis of amino acids (1.6% each) and carbon metabolism (1.5%/1.6%). Additional file 1: Figure S6 shows some important pathways for sugarcane including purine metabolism, starch and sucrose metabolism, phenylpropanoid biosynthesis (including lignin synthesis) and carbon fixation pathway.

### Comparative analysis with closely related species

**Fig. 8:**
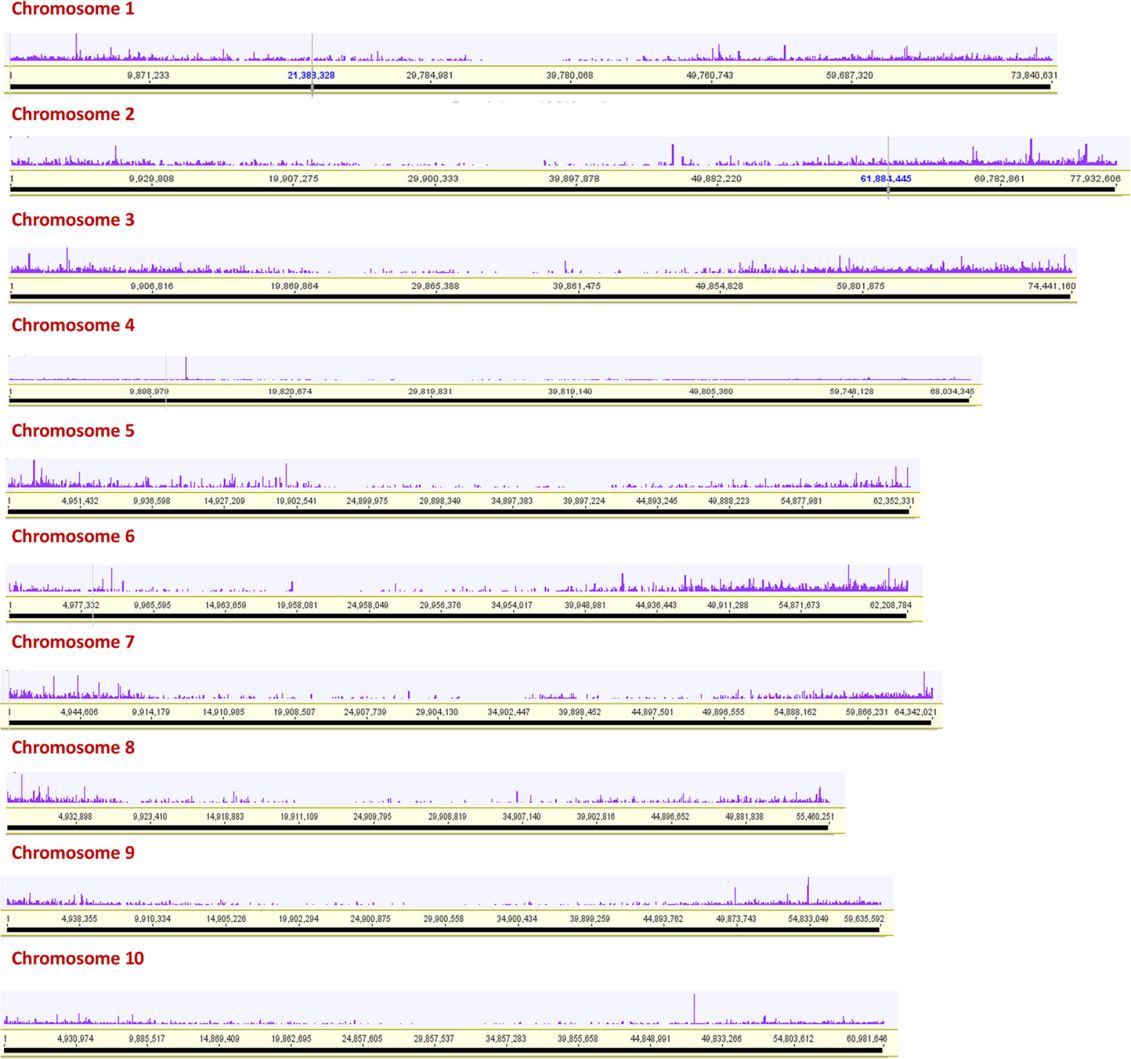
PacBio transcript isoforms aligned against the sorghum chromosomes. Purple blocks represent for the transcript isoforms distribution along the sorghum chromosomes

It was found that, 69.4% of total PacBio transcript isoforms (Fig. 8) and 41% of *de novo* contigs were aligned to the sorghum genome. When considering only retained ORFs from TransDecoder in both datasets, 80.8% of PacBio ORFs and 70% of *de novo* ORFs mapped to the sorghum genome. There were 78.7% of Evigene predicted PacBio transcripts and only 37% of the *de novo* predicted transcripts that aligned to the sorghum genome. Details are provided in Additional file 1: Table S4.

## Discussions

In the eukaryotic cell, about 95% of genes undergo RNA transcript splicing where most introns are removed and exons are retained, resulting in multiple alternative transcripts (isoforms) of the gene(s) [31, 32]. Isoforms of each gene can be formed by *cis*-splicing or *trans*-splicing, where different exons are combined together to create an mRNA molecule. In general, *cis*-splicing involves processing a single molecule [33, 34], whereas in *trans*-splicing, many pre-mRNAs are processed and their exons joined and ligated [35]. Most nuclear gene-related splicing in plants have been found to involve *cis*-splicing, in different modes such as intron retention, alternative splice or exon skipping/inclusion. For instance, in the model plant, *Arabidopsis thaliana*, it was found that about 61% of multi-exonic genes displayed alternative splicing, including different modes, ~40% intron retention, ~15.5% alternative 3’ splice site, ~8% exon skipping/inclusion and ~7.5% alternative 5’ splice site [36]. Similar proportions were reported in the transcriptomes of sorghum [28] and maize [29] with intron retention being the most abundant splicing mode, accounting for about 40%. *Trans*-splicing has been observed mostly in plant organellar genomes, such as in mitochondria [37–39] but was recently also found in maize nuclear genes [29]. Therefore, it is estimated that there are more transcripts than genes in a given genome, for example, in *Arabidopsis* cells, there are on average 300,000 transcripts from about 25,000 genes [40]. Different spliced isoforms can be translated into different proteins and could be present in the sample at different levels of expression, at different developmental stages [29]. Within the total transcript population, about 20% is comprised of high abundance transcripts of a few genes (5–10 genes), about 40-60% is from the intermediate abundance transcripts (500–2,000 genes), while 20–40% is from the rare transcripts [41, 42].

We generated an initial collection of 107,598 unique sugarcane transcript isoforms in our experiment. This transcriptome dataset provides direct evidence of alternative splicing of transcripts for each of the genes in the sugarcane genome with higher confidence, compared to alternative splicing events reported in the *de novo* assembly from short-reads. Even though PacBio offers longer reads than other current platforms (in this study, we obtained a maximum read length of 53,235 bp), it has a higher error rate [43]. In Iso-Seq, the error rate is expected to be lower since the reads are a consensus from multiple sequencing passes of the circular cDNA in the SMRT cell (PacBio). However, due to relatively low supporting reads for the low quality reads in the Quiver self-correction pipeline, it was observed that the transcript isoforms produced a large proportion of fragmented ORFs, and the transcript prediction resulted in a low number of transcripts. In a study in [28] on sorghum transcripts, it was estimated that the error rate in PacBio Iso-Seq was 2.34%, including 0.64% mismatches, 1.07% insertion (average length of 1.23 bp) and 0.63% deletion (average length of 1.16 bp). In this study, after a further error correction using the RNA-Seq reads obtained from the same experiment, the PacBio transcripts isoforms generated longer ORFs, better prediction results, better performance in completeness assessments and more reads aligned to the closely related sorghum genome. It is important to note that the RNA-Seq reads were generated from only internodal samples, while PacBio data included internode, leaf and root samples. Therefore, it is expected that there were low quality transcripts originally from leaf and root tissues, and also rare transcripts resulting from the normalization, left un-corrected in this second error correction.

Using the PacBio Iso-Seq to capture full-length transcripts without assembly overcomes the difficulty posed by the short-read data. The comparative analysis with the *de novo* assembled contigs from Illumina RNA-Seq reads, allowed us to evaluate the benefits of each of the assemblies in constructing the sugarcane transcriptome. The short-reads from the Illumina platform have been used widely for RNA-Seq differential gene expression analysis [21, 22, 44] since it provides sufficient depth and a lower error rate compared to reads generated from PacBio. However, due to the complexity of the alternative spicing mechanism of eukaryotic cells, recovering full-length transcripts has been a challenge for most of the assemblers using short reads, such as Trinity, SOAPdenovo-Trans or Velvet/OASES. Many more transcripts were generated from the *de novo* assembly in this study compared to the PacBio transcript isoforms, as well as the expected number of transcripts. This was in agreement with most *de novo* assembly studies, such as in [26], [45] and [46].

It was found that *de novo* assembly from combined multiple settings/assemblers showed that this analysis represented well the sample from which it was derived, with 98.5% of reads mapping back to transcripts, compared to 86.8% to PacBio transcript isoforms. The average proportion mapping to the reference transcriptome found in the literature is around 70-90%, i.e. in [47], since there is a proportion of reads from the lowly expressed transcripts (or low sequencing depth reads) that are not assembled into contigs. The higher read mapping rate in our *de novo* assembly could also be attributed to our library preparation, in which 150 bp paired reads were generated from a library of average 200 bp fragments, creating overlapped reads easy to assemble (an estimation of ~84% paired reads having overlapped ends could be joined into a single reads, data not shown). It could also be due to the great depth of reads used for assembly (a total 1,015,845,414 reads). Even though the PacBio data included the same internodal RNAs as *de novo* dataset, it has been through different library preparation, where the cDNAs were produced from only polyA RNAs (should be mostly mRNA and long non-coding RNAs with a polyA tail [48]). The comparison of transcripts between the two assemblies suggests that the common transcripts between the two assemblies would mostly be polyA RNAs; while the transcripts unique to PacBio (38%) could be rare transcripts that come from the normalization process and wider tissue coverage, and those unique to *de novo* assembly (33%) could be attributed to other types of RNAs in the samples. It has been proposed that *de novo* transcript contigs could be a good resource for studying the diversity of non-coding RNAs [49].

The higher CEGMA/BUSCO completeness alignment of *de novo* transcript contigs (93–97.6%) compared to PacBio transcript isoforms (83.6–96.8%) suggests that this dataset contained more expected core conserved genes, and indirectly indicates that more genes were captured. This result was also consistent with the blast search, in which more *Viridiplantae* proteins matched the *de novo* transcript contigs (24,983) than that of the PacBio transcript isoforms (18,192) at a length coverage cutoff of 70%. The *de novo* transcript contigs incorporated more gene content (especially at lower percentage of protein length coverage, Fig. 3) compared to the PacBio transcript isoforms. This could be due to the high coverage of RNA-Seq sequencing and the use of multiple settings/assemblers in our *de novo* assembly, while the sequencing depth in PacBio Iso-Seq was still relatively low.

The PacBio Iso-Seq, on the other hand, was shown to be better in recovering full-length transcript isoforms (39,999 transcript isoforms compared to 33,762 transcripts in the *de novo* transcript contigs; covered 103/164 selected genes at ≥90% coverage compared to 87/164 genes in *de novo* contigs), to include more coding transcripts (93.5% contained ORFs compared to 54.2%), and to have a much longer ORFs. Even though the number of predicted ORFs in the PacBio dataset was less than that in the *de novo* dataset, the ORFs had a longer N50 (1,158 bp) while *de novo* ORFs’ N50 was only 738 bp. Similarly, there were less predicted transcripts in the PacBio dataset than that in the *de novo* contig set. The number of predicted main transcripts (equivalent to unigenes) in the PacBio dataset was 25,012, which was approximately 71% of the total predicted genes in sugarcane (~35.000 genes). Combined with the CEGMA/BUSCO alignment, this suggests that a proportion of the genes were missing in the PacBio data. Many of these could be genes expressed in different tissues or developmental stages to those sampled here.

In the *de novo* transcript contigs, 56,766 predicted main transcripts were obtained, which was about ~162% of the predicted sugarcane genes. It could be that in *de novo* assembly, not all transcripts were recovered in full-length. There could be genes that were represented by several different contigs resulting in a total predicted transcript number greater than the true number expected. This result compares with the unigenes dataset [10], which had 79.6–90.3% CEGMA/BUSCO completeness, but resulted in only 13,205 main predicted transcripts. The unigene dataset originally had 119,768 contigs, assembled from ~445 million of 72 bp paired-end reads, of which only unigenes (representative main isoforms) were retained for further analysis. The high CEGMA/BUSCO alignment could be due to good representation of contigs for the samples, while a low number of predicted transcripts could have resulted from only the main isoforms being retained in the final contig set. Analysis of the predicted transcripts in the SoGI dataset concluded that was in agreement with the estimation reported in [2, 3] and indicates that this dataset represents ~90% of predicted sugarcane genes. Of a total 41,042 predicted transcripts by Evigene, 32,013 main transcripts were identified, which was equivalent to 91.5% of the total sugarcane predicted genes, and close to the figure above. This dataset had a low CEGMA/BUSCO alignment, ranging from 46.7 to 62.9%. It could be that the CEGMA/BUSCO alignment required 70% alignment to the conserved proteins, whereas SoGI database contained a proportion of short EST sequences (minimum 100 bp) making the alignment length less than the threshold used by the programs, and therefore resulted in low completeness. Amongst all dataset, PacBio transcript isoforms had the longest average length of the largest 1,000 proteins (1,348 aa, compared to 298 aa, 298 aa and 287 aa of *de novo* contigs, unigenes and SoGI dataset, respectively). Even though the *de novo* assembly was shown to have better completion, suggesting more gene content included, it represented fragmented sequences. The number of alternate transcripts reported for PacBio data was 26,013, and for the *de novo* dataset was 26,275, despite many more *de novo* input transcript contigs in this dataset. The PacBio predicted transcripts could be used to improve the length of sugarcane predicted gene models, and sugarcane protein sequences, which are covered by the SoGI database but are not full-length sequences.

Long non-coding RNAs are RNAs longer than 300 bp that do not encode proteins (do not have ORF ≥100 aa) and potentially play important roles in gene regulation of eukaryotic cells [48]. Their numbers, characteristics and genetic patterns in the genome still remain unclear. The prediction and annotation of long non-coding RNAs is normally challenging since unlike the coding RNAs, they are not orthologous and lack homology between closely related species. Therefore, the information from one species is not useful in non-coding RNA prediction for other species [50]. More often, long non-coding RNAs of a given genome are predicted by subjecting the un-known non-ORF-containing RNAs to a model, which is built on a set of high confidence non-coding RNAs and a partition of coding transcripts of that genome [51]. In this study, *de novo* assembly included more non-coding RNAs (45.8% of total contigs) compared to the PacBio dataset (6.5% of total isoforms). We identified 2,426 transcript sequences (accounting for 2.3% of total transcripts) as putative long-noncoding RNAs in the PacBio transcript isoforms. This was done by comparing the non-coding transcripts against the available protein and genomic databases.

## Conclusions

The transcript data generated in this study probably accounts for about 71% of the total predicted genes in the sugarcane genome. The PacBio Iso-Seq analysis recovered more full-length transcripts, with a longer N50, more ORFs and predicted transcripts and higher average length of the largest 1,000 proteins, compared to that of the *de novo* contigs from RNA-Seq. Analysis of the gene content in the two assemblies suggests that RNA-Seq covered more gene content, and more RNA classes, probably as a result of the greater sequencing depth. The majority of transcript isoforms captured in PacBio Iso-Seq were protein-coding sequences (93.5% containing ORFs ≥100 aa), whereas only 54.2% of the total RNA-Seq *de novo* contigs contained ORFs. About 92.3% and 34.7% of PacBio and *de novo* transcripts matched the *Viridiplantae* protein database, respectively. The use of normalization and the inclusion of more tissue (types/stages) in the Iso-Seq library preparation may have contributed to the recovery of the unique fraction (accounting for ~38%) attributed to rare and tissue-specific transcripts that were not covered in the RNA-Seq. Comparative analysis with the sorghum genome indicated a high content of orthologous genes between the two genomes. The total set of 51,025 predicted transcripts in the study could be used to improve sugarcane gene models and sugarcane proteins in the sugarcane databases like SoGI that lack full-length sequences. This dataset will serve as reference sequences representing full-length transcript isoforms that are expressed in sugarcane leaf, internode and root tissues, and facilitate differential expression analysis allowing exploration of different isoforms of genes to be studied. This reference database is termed as the SUGIT database (short for the SUGarcane Isoform-sequencing Transcriptome database).

## Methods

### Samples selection and preparation

**Fig. 9:**
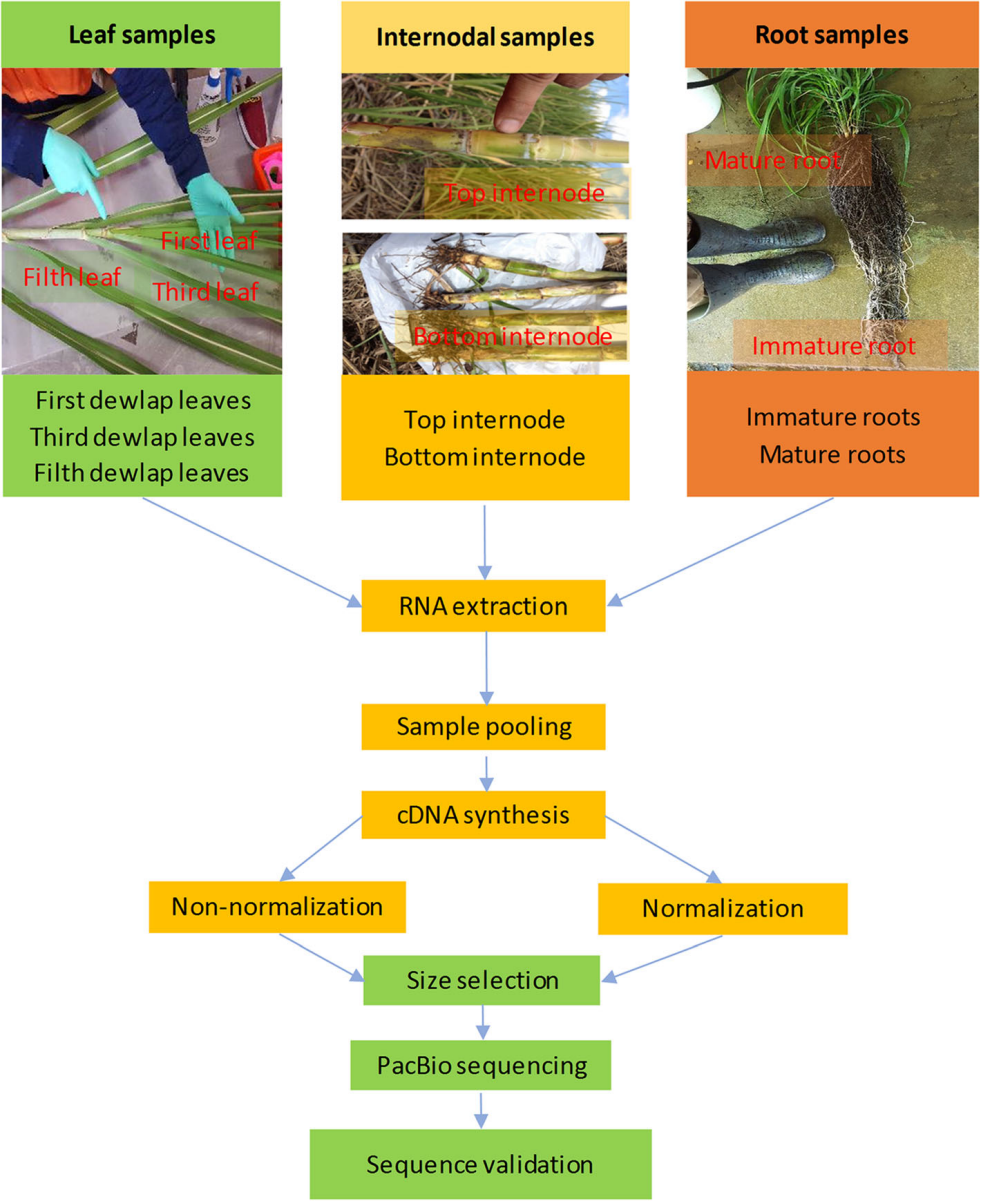
Sugarcane sample collection from leaf, internodal and root tissues used for this study

Six leaf samples, 40 internodal samples and four root samples were collected, including 22 commercial and introgressed sugarcane varieties (Table 8), provided by Sugar Research Australia (SRA), Brisbane, Australia. These samples were derived from a sugarcane population previously described in [52]. To obtain a good representation of sugarcane transcriptome, samples were collected from different developmental stages. Leaves from the first, third and fifth visible dewlap; the fourth internodes from the top and the third internode from the bottom; and immature and mature roots from immature potted sugarcane plants were collected (Fig. 9). Immature root was defined as 10 cm of the lower most root ends (containing the apical meristems and root caps), while mature root was 10 cm long from 2 cm underneath the stem crown (containing less of developing tissue). Three replicates were collected and pooled for each leaf and root stage, while four representative stalks were pooled for each internode sample. Samples were snap-frozen in liquid nitrogen within 1 min after being excised and stored at −80 °C until RNA extraction. Prior to RNA extraction, frozen samples were pulverized using a Retsch TissueLyser (Retsch, Haan, Germany) at a frequency of 30/S for 1 min 30 s. About 1 g of pulverized sample powder was used for RNA extraction.

**Table 8:** Sugarcane varieties used for RNA extraction

Sample type	Varieties	Tissue description
Leaf tissue samples	KQ228, Q208	The first, third and fifth visible dewlap leaf of mature plants
Stalk tissue samples^a^	QC02-402, QA02-1009, QN05-1460, QN05-1743, QN05-1509, QS99-2014, QA96-1749, Q241, Q200, QN05-803, KQB07-23863, KQB08-32953, KQB07-23990, KQ08-2850, KQB07-24619, KQB07-24739, QBYN04-26041, KQB09-23137, KQB09-20620, KQB09-20432	Internode 3 from the top and internode 2 from the bottom of high, medium and low fiber content sugarcane varieties from mature plants
Root tissue samples	KQ228, Q208	Mature roots and root apex from immature plants

^a^Samples were used for RNA-Seq, while all was used for PacBio Iso-Seq

### RNA extraction

RNA extractions were conducted using a two-step protocol as described in [53] employing a Trizol kit (Invitrogen), followed by a Qiagen RNeasy Plant minikit (#74134, Qiagen, Valencia, CA, United States). The RNA quality, integrity and quantity were determined by a NanoDrop8000 spectrophotometer (ThermoFisher Scientific, Wilmington, DE, USA), and on a Agilent Bioanalyser 2100 with the Agilent RNA 6000 Nano kit (Agilent Technologies, Santa Clara, CA, USA). All RNA had RIN >7.5. For PacBio Iso-Seq, two-rounds of sample pooling was carried out. At first, three pooled samples were produced by combining 4 μg each of six leaf RNA samples, 40 internodal RNA samples and four root RNA samples, respectively. Secondly, 10 μg each of three pooled samples was mixed to form one single sample, for cDNA library construction. For Illumina RNA-Seq, an indexed library of 40 internodal RNA samples was prepared and sequenced.

### PacBio isoform sequencing (Iso-Seq)

We followed the PacBio Iso-Seq Protocol using Clontech SMARTer PCR cDNA Synthesis Kit and BluePippin Size-Selection System, with modifications described below. Two cDNA libraries, with and without cDNA normalization step, were prepared on the pooled RNA sample; to ensure that the highly abundant, intermediate abundant and rare transcripts were well covered. The non-normalized library was prepared using the SMARTer PCR cDNA synthesis kit (ClonTech, Takara Bio Inc., Shiga, Japan) and KAPA HiFi PCR kit (Kapa Biosystems, Boston, USA). Approximately 1 μg of total RNA of pooled sample was subjected to a single-step of cDNA first strand synthesis by Clontech SMARTer Kit. For PCR amplification of the cDNA, a total of 18 cycles was run, using KAPA HiFi enzyme from KAPA kit. An aliquot of amplified cDNA was normalized by Trimmer-2 kit (Evrogen, Moscow, Russia), which relies on the nucleic acid hybridization [54] and unique properties of duplex-specific nuclease (DSN) isolated from Kamchatka crab [55]. The amplified double stranded cDNA was hybridized and subjected to four DSN treatments, containing 1U DSN, 0.5U DSN, 0.25U DSN and 0U DSN (control). To recover the normalized cDNA, a total of 18 PCR cycles was performed using KAPA HiFi enzyme. The cDNA treated with 1U DSN was selected as the normalized sample for sequencing. For more details of cDNA library preparation, see Additional file 2, including a detailed method, and Figure S7.

The cDNA libraries were size-fractionated according to the PacBio Iso-Seq protocol, employing the BluePinpin system (Sage Science). Four non-normalized cDNA bins (0.5-2.5 kb, 2–3.5 kb, 3–6 kb, and 5–10 kb), and two normalized cDNA bins (0.5-2.5 kb and 2–3.5 kb) were amplified separately to recover enough cDNA for sequencing (each bin required 8 μg). The size selection, PCR amplification and sequencing of six SMRT cells were conducted on a PacBio RS II instrument, at the Ramaciotti Centre for Genomics, The University of New South Wales, Australia.

### PacBio Iso-Seq data processing and read correction

The.bax.h5 file generated from SMRT sequencing was processed following the RS_IsoSeq protocol through the SMRT analysis package ver2.3.0 (PacBio), by first running the pbtranscript.py script, to separate the FL non-chimeric, non-FL and chimeric reads of interest (ROI). The chimeras, artificial concatemers and fusion genes were removed at this step. The FL, non-chimeric ROIs were determined by having the 5’ prime-, 3’ prime- adapters and a polyA tail [56]. Subsequently, adapter sequences and polyA tails were removed. Only FL, non-chimeric ROIs were kept for downstream analysis. The FL, non-chimeric sequences were clustered by Iterative Clustering for Error Correction (ICE) to generate the cluster consensus of all FL, non-chimeric and non-FL, non-chimeric sequences. This error self-correction (polishing) was performed by a quality-aware algorithm of the Quiver software, to finally obtain the FL polished consensus sequences [57]. The non-redundant polished dataset was consisted of high quality (expected accuracy ≥99%, or QV ≥ 30) and low quality transcripts (expected accuracy <99%, due to insufficient coverage or deriving from rare transcripts. More details see Additional file 2: Figure S8.

Even though, the error rate was reduced in PacBio Iso-Seq (compared to ~15% in normal PacBio sequencing [58]) by generating consensus reads from several passes of the circular cDNA, and by self-correction; the analysis of transcript prediction, transcriptome completeness, homology search against known protein database indicated that the PacBio transcript isoforms still contained significant errors. A further correction was performed by using Illumina RNA-Seq reads of the same experiment (see Table 8 and the next Section), and two other packages, proovread [59] and Long-Read De Bruijn Graph Error Correction (LoRDEC) [60]. Default parameters were applied in proovread, while parameters of -t 5 -b 200 -e 0.4 -s 3, and k-mers 21 and 25 were used in LoRDEC.

### Illumina RNA-Seq and *de novo* assembly of the short reads

About 3 μg of each of 40 internodal RNA samples (1 top and 1 bottom internodal samples from each of 20 varieties for stalk tissue samples in Table 8) was used for indexed-library preparation (average insert size of 200 bp), employing the TruSeq stranded with Ribo-Zero Plant Library Prep Kit for total RNA library (Illumina Inc.). The library was sequenced by an Illumina HiSeq4000 instrument to obtain 150 bp paired-end read data, at the Translational Research Institute, The University of Queensland, Australia. Read adapter and quality trimming was done in CLC Genomics Workbench v9.0 (CLC-GWB, CLC Bio-Qiagen, Aarhus, Denmark) with a quality score limit of <0.001 (Phred Q score ≥30), a maximum of two ambiguous nucleotides, and removing reads below 75 bp. Only paired-end reads were kept, and concatenated into one interleaved paired-end read file prior to *de novo* assembly. Reads matching sugarcane chloroplast genome (GenBank: KU214867) and sorghum mitochondrial genome (NC_008360.1) were removed using k-mer 31 in BBDuk, BBmap v36.02 [61].

The *de novo* assembly pipeline, including read digital normalization, contig assembly and clustering, were performed on non-normalized and normalized reads. Reads were normalized by the perl script insilico_read_normalization.pl (Trinity package) for Trinity; and the two-pass BBnorm tool for read error correction and normalization (BBmap), for other assemblers. A combined strategy of multiple assemblers and multiple parameters, which was shown to perform better than a single assembler/parameter approach for the transcriptome of polyploid species [62], was run on Trinity r2013-08-14 [63], CLC-GWB, Velvet/Oases v1.2.10 [64] and SOAPdenovo-Trans v1.03 [24].

Two settings of k-mer 25 and k-mer 31 were applied in Trinity. Multiple combinations of word sizes (25, 35, 45, 55 and 64) and bubble sizes (200, 1,200 and 2,200); fast setting and no scaffolding, were employed in CLC-GWB. To remove the redundancy, all CLC-GWB derived contigs were pooled and clustered using CD-HIT-EST v4.6.5 [65] with 95% identity, to obtain one representative assembly. In Velvet/Oases, multiple k-mers from 25 to 125 with a step size of 10 were applied using the velveth to generate roadmaps of reads, then a merged assembly of preliminary contigs was formed by using the velvetg at each k-mer. Another run using velveth of k-mer 27 and velvetg (conserving long contigs) on the pooled contigs of all k-mers was run to generate the velvet merged assembly. The merged assembly was clustered by Oases, using the following settings: −merge yes -cov_cutoff 5 -edgeFractionCutoff 0.01 -min_trans_lgth 300. Likewise, a k-mer range of 25 to 125 with a step size of 10 were used in SOAPdenovo-Trans. The resultant contigs were clustered by CD-HIT-EST with 95% identity. Finally, all four assembler-derived assemblies were pooled together and clustered by CD-HIT-EST with 95% identity, to obtain the RNA-Seq *de novo* assembly.

### Read mapping analysis

To make a preliminary assessment of the transcript composition captured in the assemblies, and determine how well the transcripts represent the samples, all RNA-Seq reads were mapped back to each of assemblies. The setting of length fraction (1.0), similarity fraction (0.8), mismatch cost (2), gap (insertion and deletions) cost (3) was used in CLC-GWB. The percentage of reads aligned to each assembly was used for comparison of assemblies.

### Transcriptome completeness analysis

A protein set from 248 ultra-conserved Core Eukaryotic Conserved Genes (CEGs) [66] was employed in CEGMA (Core Eukaryotic Genes Mapping Approach) v2.5 [67], while a set of selected 956 *Plantae* conserved orthologous proteins was used in BUSCO (Benchmarking Universal Single-Copy Orthologs) v1.21 [68], to assess the completeness of the conserved content in the transcriptome assemblies. The percentage of transcripts that fully aligned (≥70%), partially aligned to the conserved proteins, percentage missing proteins, were obtained and compared.

### Counting the full length transcripts

The assembly was compared against the *Viridiplantae* protein database [69] using BLASTX (BLAST+ v2.3.0) with an e-value ≤1e-20, and only the best hit was considered. The number of transcripts that appear to be FL or nearly FL (having ≥90% and ≥70% alignment coverage to known proteins) were counted using the Perl script analyze_blastPlus_topHit_coverage.pl from Trinity package, and compared between the assemblies. Additionally, we adopted this method in using a set of 164 selected genes from sugarcane and other grass species (see Additional file 1: Table S5) to assess the ability in capturing these genes in FL in the assembly. The presence and alignment of these known genes were used further in assessing the quality of the assemblies.

### Open reading frame and coding potential analysis

The open reading frames (ORFs) were detected by using the package TransDecoder [70] with a minimum length of 100 amino acid (aa), a log-likelihood score to each of six reading frames, and multiple ORFs were allowed to be reported for a single transcript. The candidate coding regions (the longest ORF amongst the overlapped frames) were extracted by transDecoder.LongOrfs. The candidate ORFs were subjected to homology search against the Pfam protein domain database, using HMMER v3.1b2 [71]; and the UniProt *Viridiplantae* protein database using BLASTP (BLAST+ v2.3.0), e-value ≤1e-5. Finally, all candidate ORFs with the Pfam domain and BLASTP hits were retained by transDecoder.Predict module.

Alternatively, the tr2aacds pipeline (EvidentialGene package v2013.07.27, Evigene [72, 73]) was used to predict the best main and alternate transcripts from the potential coding sequences in the assemblies. The sequences then were clustered based on the amino acid sequences generated to remove the redundancy and for the best coding amongst each of the clusters by exonerate-2.2.0 [74], CD-HIT-EST (with 100% identity) and BLASTN (BLAST+ v2.3.0), (with an e value of 1e-19). The total CDS sequences, total predicted transcripts (main and alternate), the % dropped CDS and the average length of 1,000 longest proteins were used to compare between the assemblies.

### Characterization of non-coding RNAs

We filtered out the sequences with an ORF ≥100 aa, after which the remaining sequences were characterized for potential non-coding RNAs including small RNAs, microRNAs and long non-coding RNAs (lncRNAs). The proportion of the candidate long non-coding RNAs, which were ≥ 300 bp and did not exhibit ORF ≥100 aa, were compared between assemblies.

### Repeat content analysis

The transposable element (TE) domains present in the data were identified by RepeatMasker v4.0.6 [75], using the sensitive search engine: cross_match v1.090518 [76], repbase complete database release 20150807 [77], RepeatMasker database v20150807 and Dfam 2.0 library [78]. A customized repeat library was built, including 174 ancestral and ubiquitous sequences; and 7,851 linear specific sequences for *Viridiplantae*.

The MISA program v1.0 [79] was used to detect the simple sequence repeats (SSRs) in the assemblies. Only the motifs having two to six nucleotides were considered, and a sequence with two or more SSRs with maximum interrupted length of 100 bp was considered as SSRs present in compound formation.

### Transcript annotation

Transcript sequences were compared against the NCBI NR nucleotide database (BLASTN), the *Viridiplantae* proteins, sorghum proteins [80] (BLASTX), and sugarcane EST database [81] (BLASTN), at an e-value ≤1e-5. Gene names were assigned to the highest scored hit.

The data was compared against the sugarcane chloroplast genome (GenBank: KU214867), sorghum mitochondrial genome (GenBank: NC_008360.1) and maize mitochondrial genome (GenBank: NC_007982), to detect the chloroplast and mitochondrial genes captured in assemblies. The transcription factor (TF)-encoding transcripts were annotated by comparing against the plant transcription factor database (PlantTFDB v3.0) [82] and sugarcane transcription factor database (9,672 TFs from 48 TF families) [83].

Further transcript functions were annotated by RunIproScan v1.1.0 [84], using InterProScan-5.19-58.0 [85], mapping against the known protein domain database, ORF ≥30 aa. GO terms were enriched and plotted by WEGO [86]. Kyoto Encyclopedia of Genes and Genomes (KEGG) pathway mapping was done on the KEGG Automatic Annotation Server (KAAS) v2.0 [87], taking all plant species as references, GHOSTX and bi-directional best hit method.

### Comparative analysis with closely related species

Transcripts were aligned to sorghum genome v2.0 [80] using GMAP (genome mapping and alignment program) [88] with 80% identity and 90% coverage threshold to compare between the two genomes. SAMtools v1.2 [89] were used to analyse the mapping files.

### Data analysis

Assemblies were assessed by QUAST program [90]. All Venn diagrams were created by the online Venn tool [91]. All analyses using command-line packages were performed at the High Performance Computer clusters, Euramoo, Flashlite and Tinaroo, hosted by Research Computing Center, The University of Queensland, Australia [92]. The CLC-GWB analyses were conducted on a CLC Genomics Server the CLC server, nodes and CLC-clients which are part of the Robert Henry Bioinformatics infrastructure at QAAFI, the University of Queensland, Australia.

## Additional files

Additional file 1: Figures S1. Length (bp) distribution of all PacBio Iso-Seq reads of inserts (ROIs). **Figure S2** QC report of sugarcane RNA-Seq reads. **Figure S3** Summary statistics of sugarcane *de novo* assembly. a, Summary statistics by QUAST. b, Cumulative length. c, Contig length distribution. d, GC content. **Figure S4** Length (bp) distribution of 2,426 candidate long non-coding RNAs in the sugarcane transcriptome. **Figure S5** Distribution of the transcription factor families in the sugarcane transcriptome captured by PacBio Iso-Seq. The values are expressed in percentage (%) of total TFs detected. **Figure S6** Important KEGG pathways in sugarcane. a, Purine metabolism. b, Starch and sucrose metabolism. c, Phenylpropanoid biosynthesis (including lignin synthesis). d, Carbon fixation pathway. The highlighted boxes represents PacBio transcript isoforms annotated against the KEGG metabolic pathway. **Table S1** Correction of sugarcane PacBio transcript isoform data using Illumina short-reads. **Table S2** Repeat content masking analysis of sugarcane transcriptome. **Table S3** Simple sequence repeat annotation of sugarcane transcriptome. **Table S4** Sugarcane transcripts aligned against the sorghum genome. **Table S5** List of 164 selected genes from sugarcane and grass family used in the full-length assessment. (PDF 2918 kb)

Additional file 2 Additional Method: More details of PacBio Iso-Seq library preparation and data processing. **Figure S7** Non-normalized and normalized cDNA libraries used in this study. a, RNA profiles of individual sample pooling and all sample pooling in Bioanalyser. b, Two non-normalized samples on agarose 1.2%, mouse RNA was used as control. c, Results of normalized cDNA samples treated with 1U and 0.5U DSN. d, A comparison between the non-normalized and normalized cDNA profiles. **Figure S8** PacBio Iso-Seq data processing and read correction (adopted from Pacific Biosciences). (PDF 669 kb)

## Abbreviations

BUSCO: Benchmarking universal single-copy orthologs
cDNA: complementary DNA
CDS: Coding sequence
CEGMA: Core eukaryotic genes mapping approach
CLC-GWB: CLC genomics workbench
DSN: Duplex-specific nuclease
EST: Expressed sequence tag
FL: Full-length
GMAP: Genome mapping and alignment program
GO: Gene ontology
Iso-Seq: Isoform sequencing
KEGG: Kyoto encyclopedia of genes and genomes
KO: KEGG orthology
LINEs: Long interspersed nuclear elements
LTR: Long terminal repeat
NR: Non-redundant
ORF: Open reading frame
PCR: Polymerase chain reaction
QC: Quality control
RIN: RNA integrity number
RNA-Seq: RNA-Sequencing
ROI: Reads of inserts
SINEs: Retroelements included short interspersed nuclear elements
SoGI: *Saccharum officinarum* gene indices
SRA: Sugar Research Australia
TAPIS: Transcriptome analysis pipeline for isoform sequencing
TE: Transposable element
TF: Transcription factor
