# Supplementary material for: A survey of the complex transcriptome from the highly polyploid sugarcane genome using full-length isoform sequencing and de novo assembly from short read sequencing
Source: BMC Genomics. 2017 May 22;18:395. doi: 10.1186/s12864-017-3757-8 (PMC5440902; doi:10.1186/s12864-017-3757-8)
Supplement: Supplementary file 1 — Length (bp) distribution of all PacBio Iso-Seq reads of inserts (ROIs). Figure S2 QC report of sugarcane RNA-Seq reads. Figure S3 Summary statistics of sugarcane de novo assembly. a, Summary statistics by QUAST. b, Cumulative length. c, Contig length distribution. d, GC content. Figure S4 Length (bp) distribution of 2,426 candidate long non-coding RNAs in the sugarcane transcriptome. Figure S5 Distribution of the transcription factor families in the sugarcane transcriptome captured by PacBio Iso-Seq. The values are expressed in percentage (%) of total TFs detected. Figure S6 Important KEGG pathways in sugarcane. a, Purine metabolism. b, Starch and sucrose metabolism. c, Phenylpropanoid biosynthesis (including lignin synthesis). d, Carbon fixation pathway. The highlighted boxes represents PacBio transcript isoforms annotated against the KEGG metabolic pathway. Table S1 Correction of sugarcane PacBio transcript isoform data using Illumina short-reads. Table S2 Repeat content masking analysis of sugarcane transcriptome. Table S3 Simple sequence repeat annotation of sugarcane transcriptome. Table S4 Sugarcane transcripts aligned against the sorghum genome. Table S5 List of 164 selected genes from sugarcane and grass family used in the full-length assessment. (PDF 2918 kb) [file 12864_2017_3757_MOESM1_ESM.pdf]

Figure S1. Length (bp) distribution of all PacBio Iso-Seq reads of inserts (ROIs)

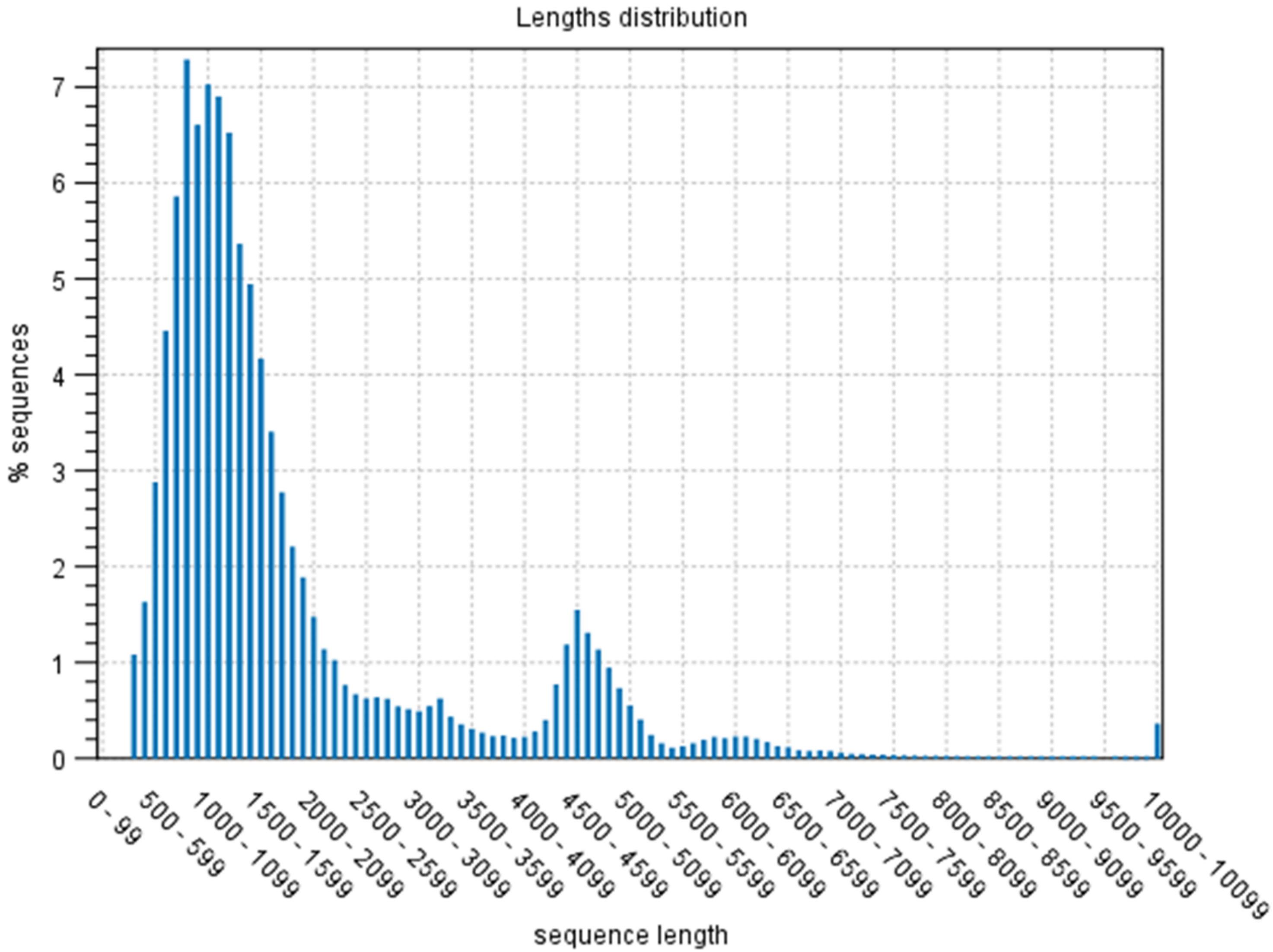

Table S1. Correction of sugarcane PacBio transcript isoform data using Illumina short-reads

	Analysis	PacBio non-corrected	LoRDEC non-normalized reads	LoRDEC BBnorm normalized reads	LoRDEC Trinity normalized reads	Proovread BBnorm normalized reads	Proovread Trinity normalized reads
	**Total transcripts**	107,604	107,599	107,599	107,598	107,599	107,597
**Evigene** prediction	**Okay transcripts**	**18,190**	**44,875**	**45,012**	**51,025**	**15,412**	**15,198**
	% Okay transcripts	14.6	40.6	40.4	42.9	14.5	14.6
	Drop transcripts	105,607	65,635	66,391	67,730	90,157	88,371
	% Drop transcripts	85.3	59.3	59.5	57.0	85.4	85.3
	Length 1K proteins*	385	1,340	1,339	1,348	370	368
	**Main transcripts**	**14,124**	**24,965**	**25,003**	**25,012**	**11,260**	**11,115**
	**Alternate transcripts**	**4,066**	**19,910**	**19,979**	**26,013**	**4,152**	**4,083**
	Min all set	227	186	186	186	222	227
	Max all set	9,633	8,142	8,142	8,142	4,772	4,449
	Mean all set	654	1,162	1154.2	1,180.8	235	660.1
	GC%	61.80	52.00	52.00	51.40	61.70	61.90
**CEGMA alignment**	Complete	230	240	239	240	237	237
	%Completeness	**92.74**	**96.77**	**96.37**	**96.77**	**95.56**	**95.56**
	Partial	239	243	243	243	242	242
	%Completeness	**96.4**	**97.98**	**97.98**	**97.98**	**97.58**	**97.58**
**BUSCO notation**	Complete BUSCOs	721	800	794	799	798	802
	%Completeness	**75.4**	**83.7**	**83.1**	**83.6**	**83.5**	**83.9**
	Fragmented BUSCOs	113	58	63	64	66	65
	%Fragmented	11.82	6.07	6.59	6.69	6.90	6.80
	Missing BUSCOs	122	98	99	93	92	89
	%Missing	12.76	10.25	10.36	9.73	9.62	9.31
	Complete+partial (%)	**87.13**	**89.69**	**89.64**	**90.27**	**90.38**	**90.69**
**ORFs detected**	Minimum 100 aa	243,637			252,491		
	ORF N50 (bp)	570			888		
**Proteins****	Full-length ≥90%	9.727			12,611		
**Sorghum**	%Transcripts mapped	66.43			69.44		

**Average length of largest 1,000 proteins. ** Viridiplantae protein counts which were covered ≥90% by transcript isoforms*

Figure S2. QC report of sugarcane RNA-Seq reads

QC report of sugarcane RNA-Seq reads

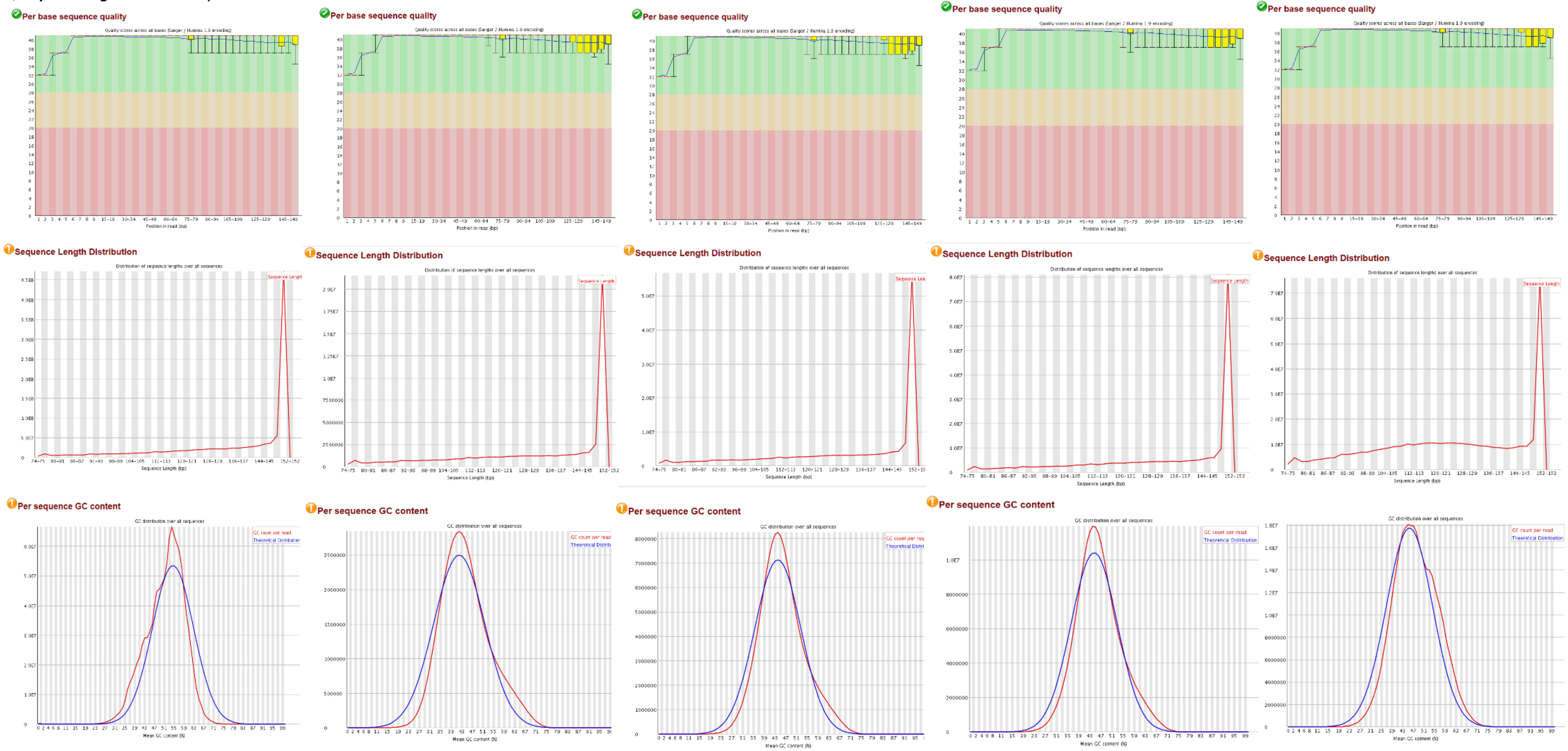

All reads trimmed Q30, min length 75bp

Trinity normalized reads (max cov 50)

Trinity normalized reads (max cov 400)

Trinity normalized reads (max cov 2000)

BBnorm normalized reads (max cov 10,000)

Figure S3. Summary statistics of sugarcane *de novo* assembly. a, Summary statistics by QUAST. b, Cumulative length. c, Contig length distribution. d, GC content.

Summary statistics of *de novo* assembly

a

Assembly	Trinity	CLC-GWB	Velvet_OASES	SOAP-Trans	All clustered
# contigs (>= 0 bp)	431,255	508,239	798,345	289,705	906,566
# contigs (>= 1000 bp)	210,220	109,992	37,698	34,781	294,867
# contigs (>= 2000 bp)	104,013	34,970	2,633	4,817	130,095
# contigs (>= 3000 bp)	46,942	13,732	441	1,099	57,437
# contigs (>= 4000 bp)	19,218	5,574	94	282	23,416
# contigs (>= 5000 bp)	7,542	2,413	31	104	9,227
Total length (>= 0 bp)	608,060,518	419,587,279	409,817,309	182,675,172	966,867,516
Total length (>= 1000 bp)	488,236,866	212,086,967	51,074,376	52,375,612	646,818,455
Total length (>= 2000 bp)	334,191,937	108,823,958	6,747,428	12,990,413	412,768,843
Total length (>= 3000 bp)	194,956,948	57,587,009	1,627,107	4,183,855	235,893,013
Total length (>= 4000 bp)	99,755,591	29,665,550	461,757	1,434,432	119,115,268
Total length (>= 5000 bp)	48,128,238	15,704,441	189,408	651,385	56,369,155
# contigs	431,255	508,239	798,345	289,705	906,566
Largest contig	28,461	29,928	11,272	18,497	9,990
Total length	608,060,518	419,587,279	409,817,309	182,675,172	966,867,516
GC (%)	43.88	43.50	42.84	43.25	43.67
N50	2,194	1,014	516	674	1,671
N75	1,216	542	389	455	745
L50	89,618	107,715	266,636	82,953	168,723
L75	181,071	253,430	497,065	165,967	385,929
# N's per 100 kbp	0	0	0	0	0

Cumulative length

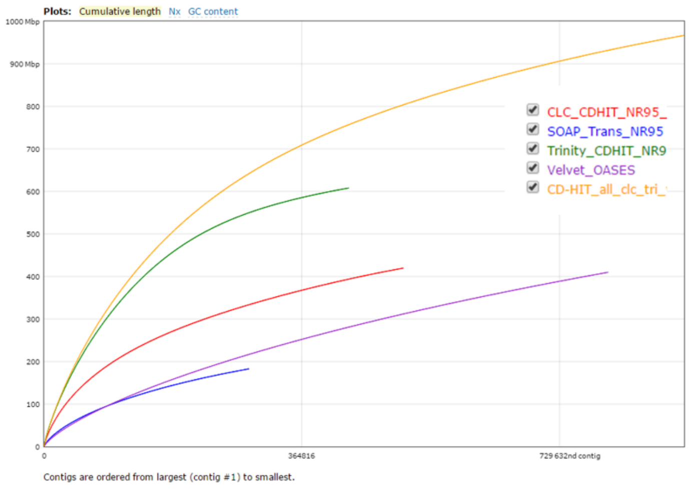

c **Nx**

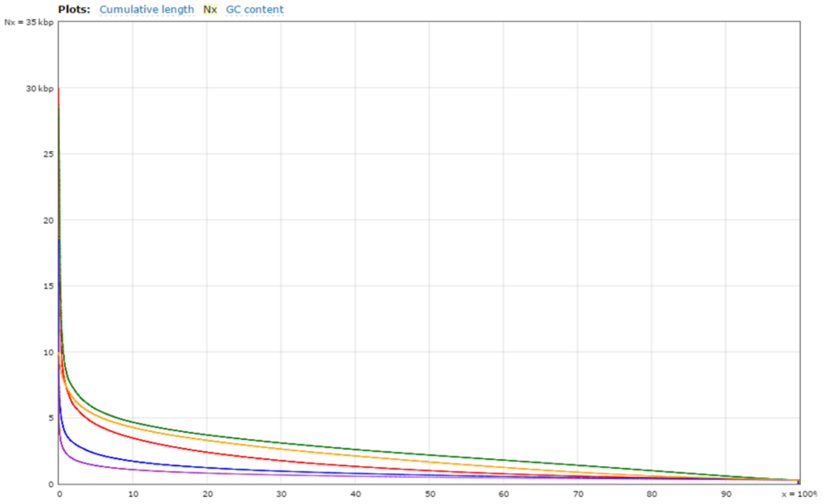

d **GC content**

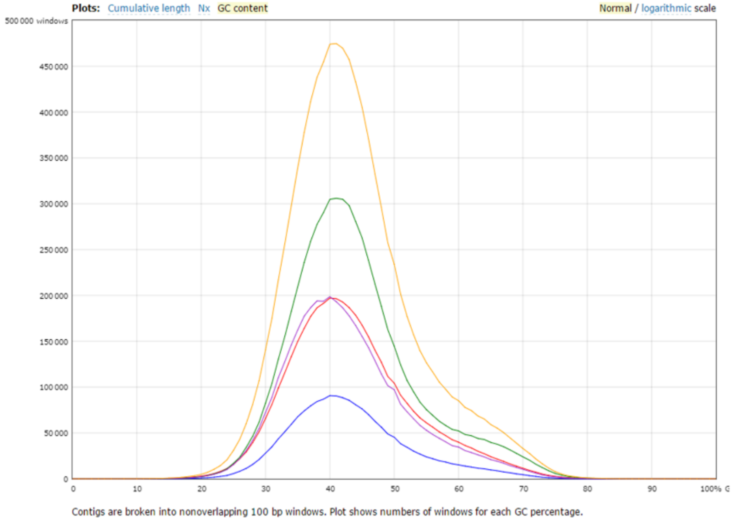

Figure S4. Length (bp) distribution of 2,426 candidate long non-coding RNAs in the sugarcane transcriptome

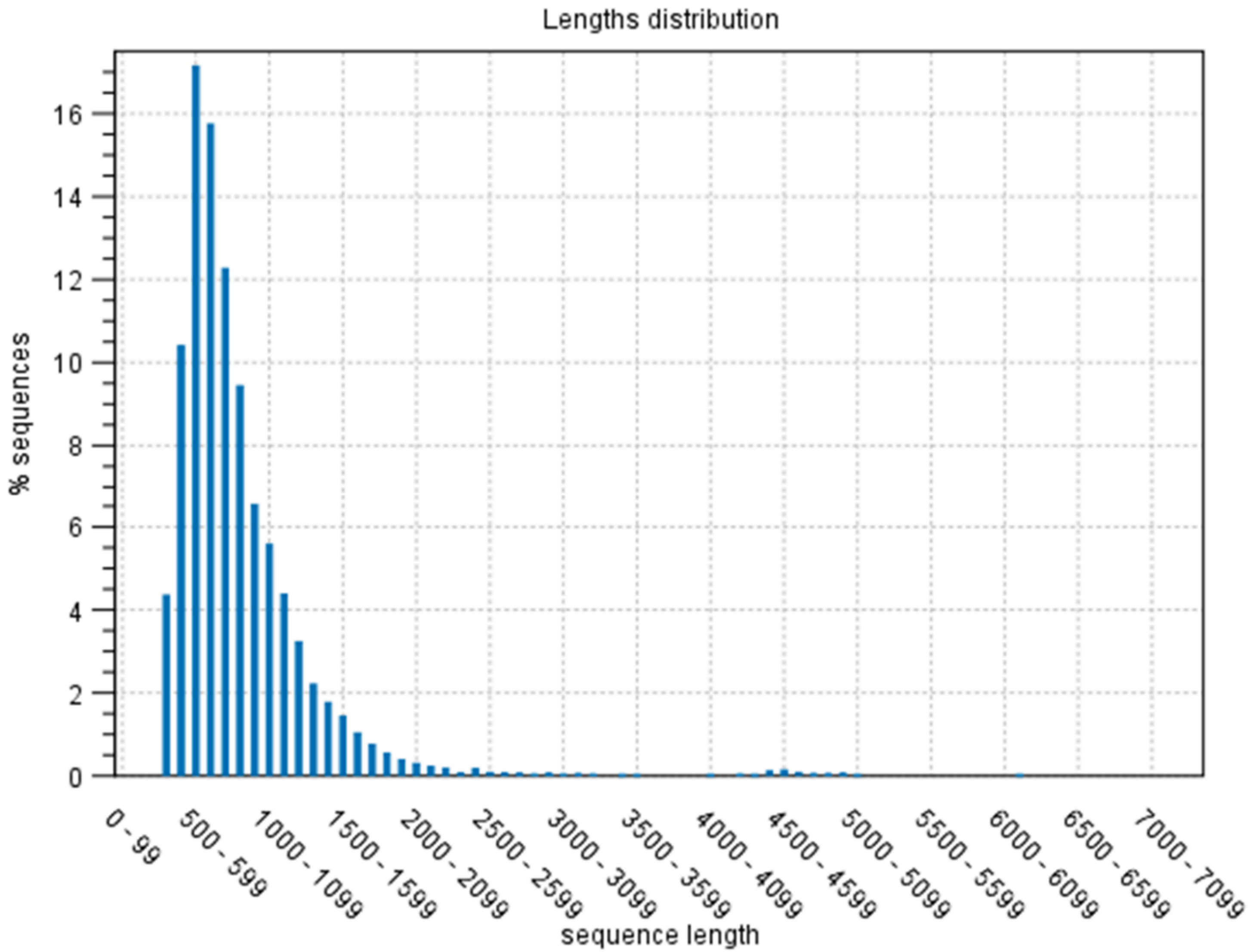

Table S2. Repeat content masking analysis of sugarcane transcriptome

Repeat class		PacBio transcript isoforms			*De novo* transcript contigs		
		Count	Length (bp)	%	Count	Length (bp)	%
**Retroelements**		**15,175**	**6,074,781**	**50.18**	**145,929**	**46,596,625**	**45.99**
SINEs:		454	61,381	1.50	7,053	930,899	2.22
Penelope		0	0	0.00	5	311	0.00
**LINEs:**		**4,186**	**2,300,430**	**13.84**	**41,531**	**11,636,323**	**13.09**
	R2/R4/NeSL	8	358	0.03	0	0	0.00
	RTE/Bov-B	959	675,286	3.17	12,971	1,589,788	4.09
	L1/CIN4	3,158	1,609,750	10.44	28,508	10,037,067	8.98
**LTR elements:**		**10,535**	**3,712,970**	**34.83**	**97,345**	**34,029,403**	**30.68**
	Ty1/Copia	3,814	1,381,035	12.61	39,631	13,429,751	12.49
	Gypsy/DIRS1	5,417	2,152,682	17.91	55,279	20,133,542	17.42
**DNA transposons**		**12,433**	**2,417,337**	**41.11**	**155,237**	**29,577,673**	**48.92**
hobo-Activator		2,102	515,214	6.95	20,904	4,755,730	6.59
Tc1-IS630-Pogo		1,434	278,049	4.74	29,821	4,459,028	9.40
Tourist/Harbinger		2,943	493,608	9.73	48,008	7,913,158	15.13
Other (Mirage, P-element, Transib)		5	307	0.02	1	66	0.00
**Unclassified:**		**2,635**	**488,879**	**8.71**	**16,139**	**3,581,084**	**5.09**
**Total interspersed repeats:**		30,243	8,980,997		317,305	79,755,382	

Table S3. Simple sequence repeat annotation of sugarcane transcriptome

	**PacBio transcript isoforms**		***De novo* transcript contigs**	
Total number of identified SSRs:	15,715		52,847	
Number of SSR containing sequences:	13,356		48,091	
Number of sequences containing more than 1 SSR:	1,906		4,304	
Number of SSRs present in compound formation:	844		2,276	
**SSR size (bp)**	**Number of SSRs**	**%**	**Number of SSRs**	**%**
2	4,250	27.04	21,702	41.07
3	10,441	66.44	27,863	52.72
4	538	3.42	2,226	4.21
5	262	1.67	655	1.24
6	224	1.43	401	0.76

Figure S5. Distribution of the transcription factor families in the sugarcane transcriptome captured by PacBio Iso-Seq. The values are expressed in percentage (%) of total TFs detected.

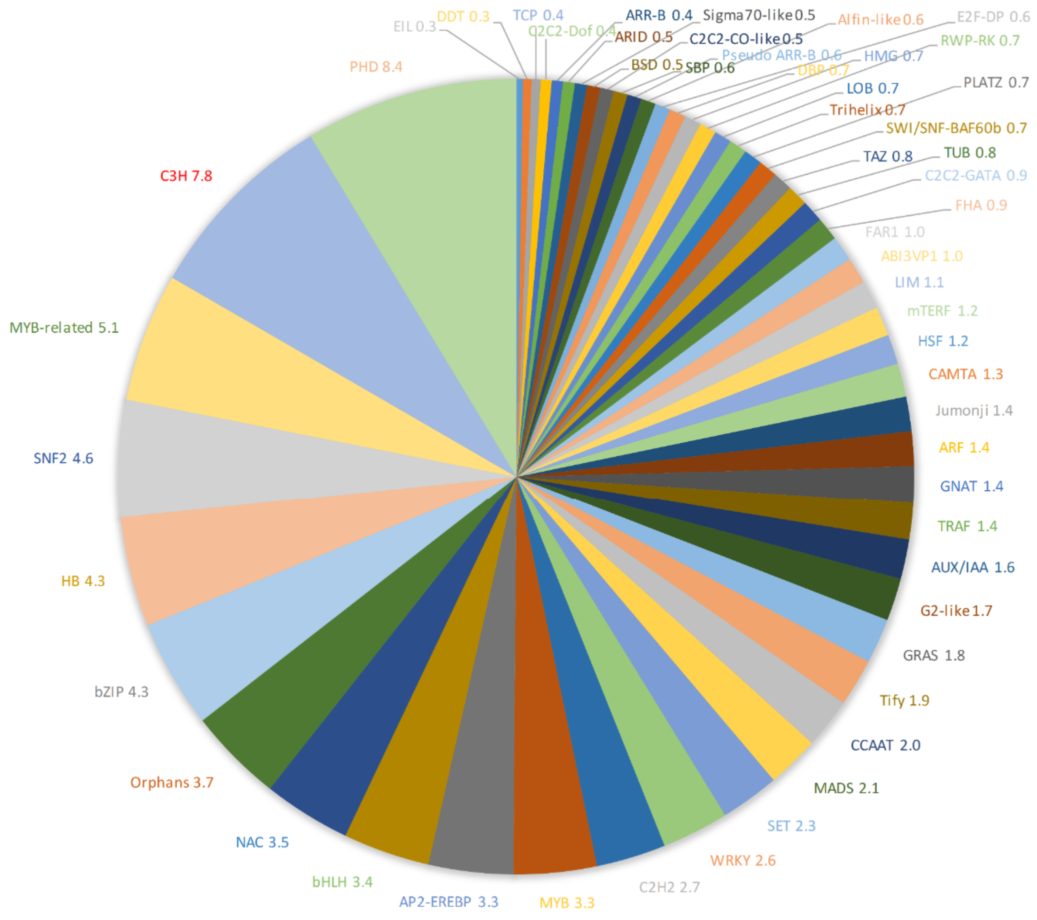

Distribution of the transcription factor families in the sugarcane transcriptome (%)

Figure S6. Important KEGG pathways in sugarcane. a, Purine metabolism. **b**, Starch and sucrose metabolism. **c**, Phenylpropanoid biosynthesis (including lignin synthesis). **d**, Carbon fixation pathway. The highlighted boxes represents PacBio transcript isoforms annotated against the KEGG metabolic pathway.

a

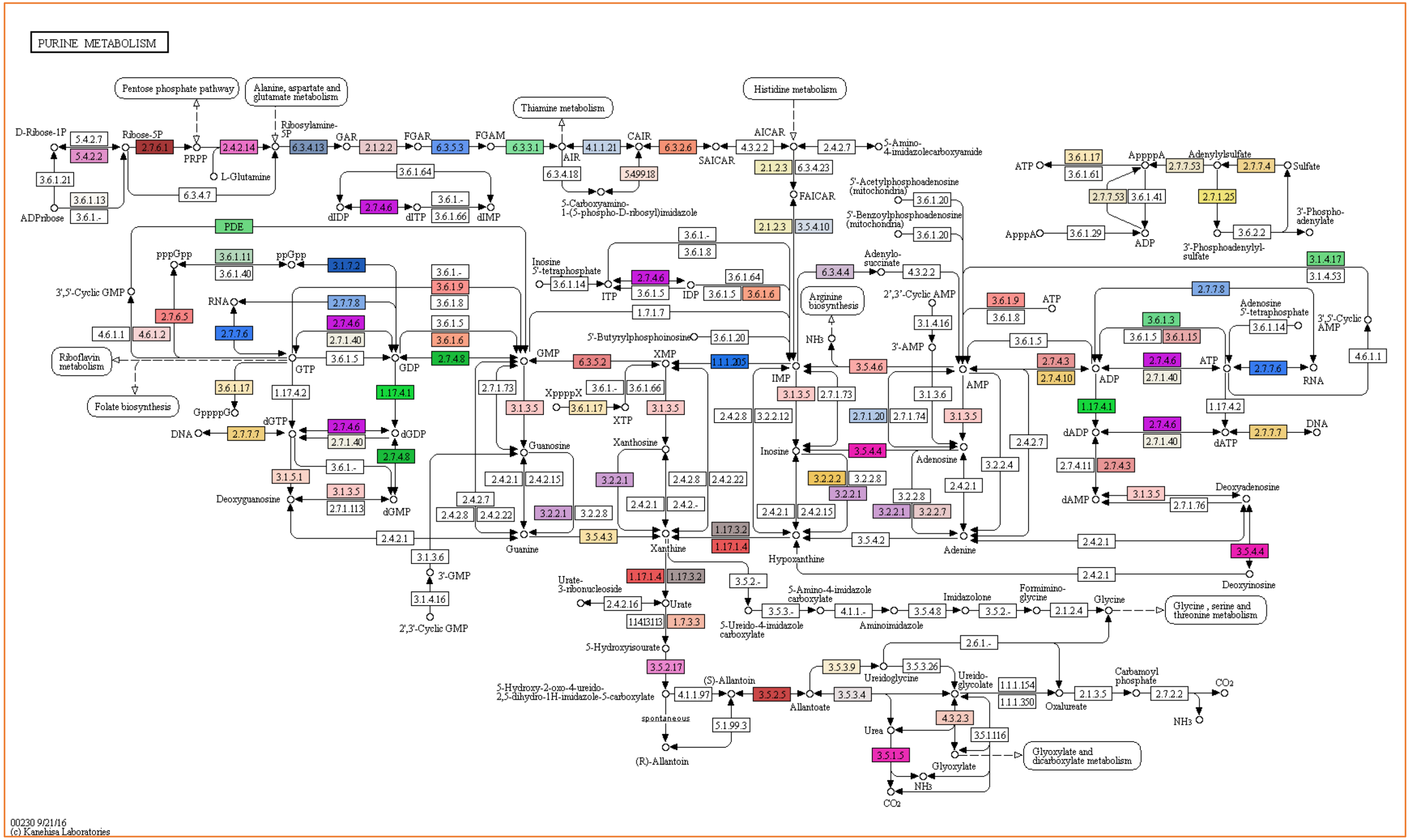

b

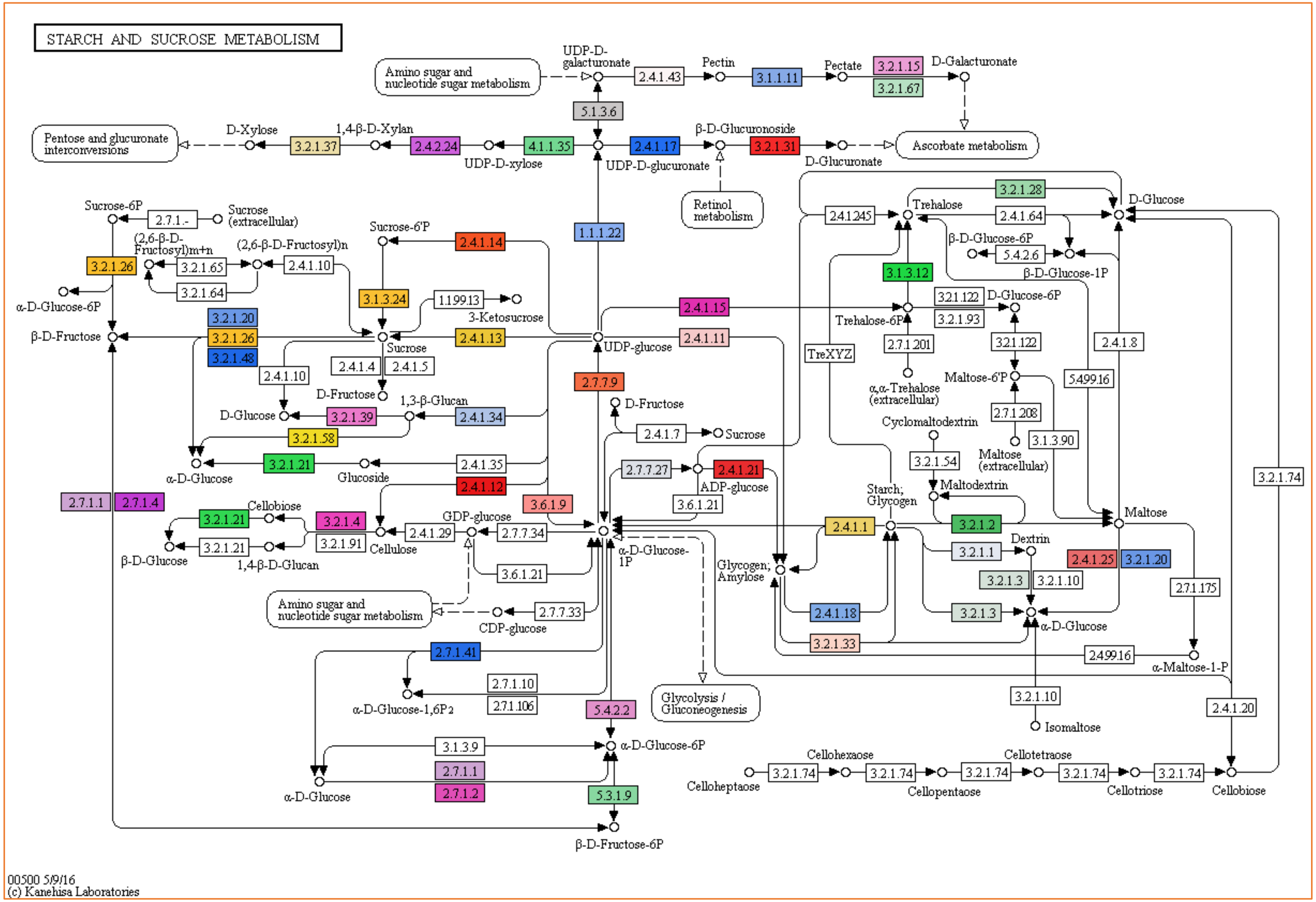

STARCH AND SUCROSE METABOLISM
Amino sugar and nucleotide sugar metabolism
UDP-D-galacturonate
2.4.1.43
Pectin
3.1.1.11
Pectate
3.2.1.15
3.2.1.67
D-Galacturonate
5.1.3.6
Pentose and glucuronate interconversions
D-Xylose
3.2.1.37
1,4-β-D-Xylan
2.4.2.24
4.1.1.35
UDP-D-xylose
2.4.1.17
β-D-Glucuronoside
3.2.1.31
UDP-D-glucuronate
D-Glucuronate
Ascorbate metabolism
Retinol metabolism
Sucrose-6P
2.7.1.-
Sucrose (extracellular)
(2,6-β-D-Fructosyl)m+n
3.2.1.65
(2,6-β-D-Fructosyl)n
2.4.1.10
3.2.1.26
3.2.1.64
α-D-Glucose-6P
Sucrose-6'P
2.4.1.14
1.1.1.22
3.1.3.24
1.1.99.13
3-Ketosucrose
3.2.1.20
3.2.1.26
3.2.1.48
β-D-Fructose
Sucrose
2.4.1.13
2.4.1.15
2.4.1.11
UDP-glucose
2.4.1.10
2.4.1.4
2.4.1.5
D-Fructose
1,3-β-Glucan
D-Glucose
3.2.1.39
2.4.1.34
3.2.1.58
2.7.7.9
D-Fructose
2.4.1.7
Sucrose
3.2.1.21
α-D-Glucose
Glucoside
2.4.1.35
2.4.1.12
2.7.7.27
2.4.1.21
ADP-glucose
3.6.1.21
3.6.1.9
2.7.1.1
2.7.1.4
Cellobiose
3.2.1.21
3.2.1.4
GDP-glucose
2.4.1.29
2.7.7.34
3.2.1.21
3.2.1.91
Cellulose
β-D-Glucose
1,4-β-D-Glucan
3.6.1.21
α-D-Glucose-1P
Amino sugar and nucleotide sugar metabolism
2.7.7.33
CDP-glucose
2.7.1.41
2.7.1.10
α-D-Glucose-1,6P2
2.7.1.106
5.4.2.2
Glycolysis / Gluconeogenesis
3.1.3.9
α-D-Glucose-6P
2.7.1.1
2.7.1.2
5.3.1.9
α-D-Glucose
β-D-Fructose-6P
3.2.1.28
Trehalose
D-Glucose
2.4.1.245
2.4.1.64
β-D-Glucose-6P
5.4.2.6
β-D-Glucose-1P
3.1.3.12
3.2.1.122
D-Glucose-6P
3.2.1.93
Trehalose-6P
2.7.1.201
3.2.1.122
2.4.1.8
TreXYZ
5.4.99.16
α,α-Trehalose (extracellular)
Maltose-6'P
2.7.1.208
Cyclomaltodextrin
3.1.3.90
3.2.1.54
Maltose (extracellular)
Maltodextrin
Starch; Glycogen
2.4.1.1
3.2.1.2
Maltose
3.2.1.74
Dextrin
3.2.1.1
2.4.1.25
3.2.1.20
Glycogen; Amylose
3.2.1.3
3.2.1.10
2.7.1.175
2.4.1.18
3.2.1.3
α-D-Glucose
3.2.1.33
2.4.99.16
α-Maltose-1-P
3.2.1.10
2.4.1.20
Isomaltose
Cellohexaose
Cellotetraose
3.2.1.74
3.2.1.74
3.2.1.74
3.2.1.74
3.2.1.74
Celloheptaose
Cellopentaose
Cellotriose
Cellobiose
00500 5/9/16
(c) Kanehisa Laboratories

c

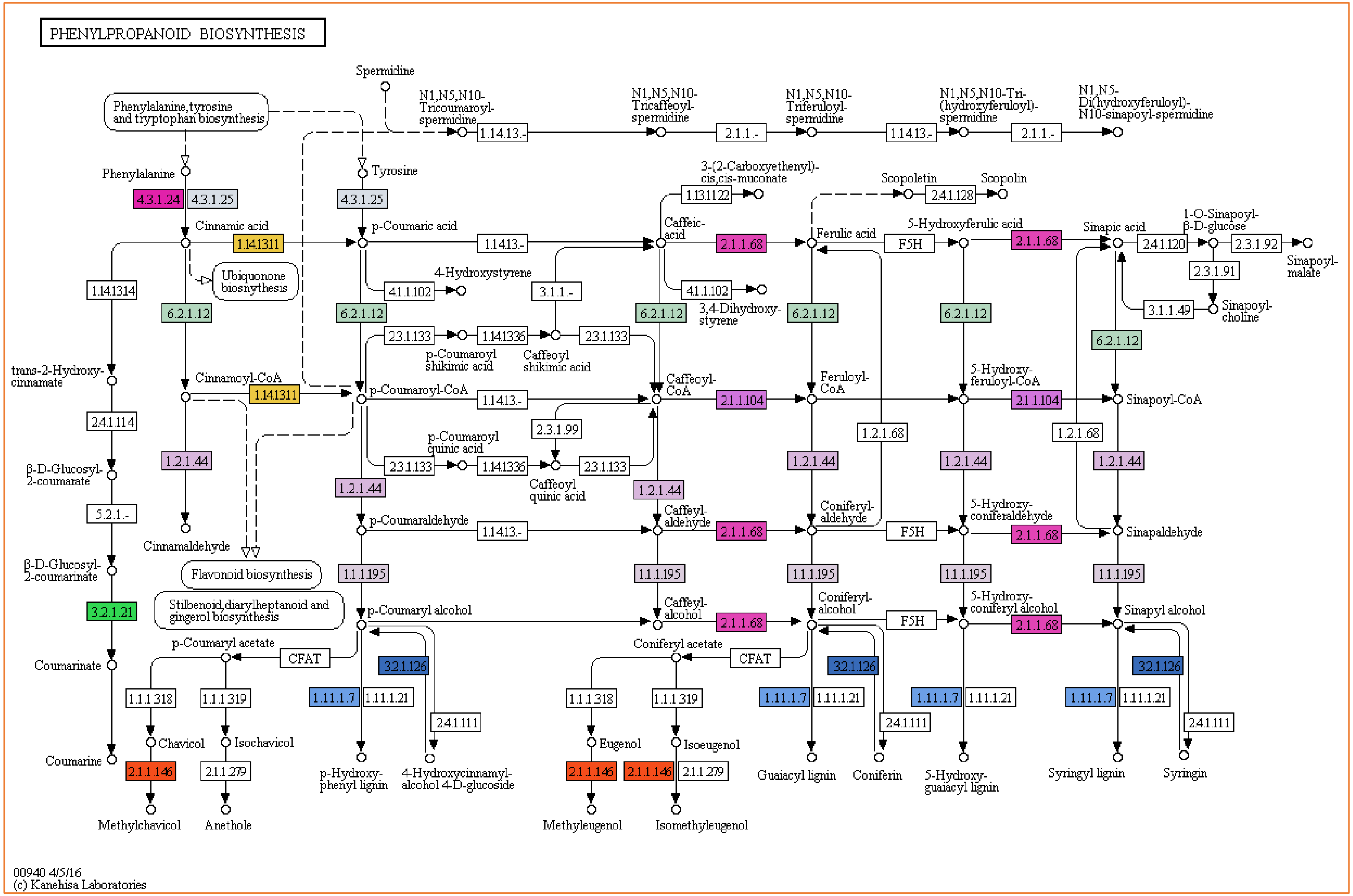
PHENYLPROPANOID BIOSYNTHESIS
Spermidine
Phenylalanine,tyrosine and tryptophan biosynthesis
N1,N5,N10-Tricoumaroyl-spermidine
1.14.13.-
N1,N5,N10-Tricaffeoyl-spermidine
2.1.1.-
N1,N5,N10-Triferuloyl-spermidine
1.14.13.-
N1,N5,N10-Tri-(hydroxyferuloyl)-spermidine
2.1.1.-
N1,N5-Di(hydroxyferuloyl)-N10-sinapoyl-spermidine
Phenylalanine
Tyrosine
4.3.1.24
4.3.1.25
4.3.1.25
3-(2-Carboxyethenyl)-cis,cis-muconate
1.13.11.22
Scopoletin
2.4.1.128
Scopolin
Cinnamic acid
1.14.13.11
p-Coumaric acid
1.14.13.-
Caffeic-acid
2.1.1.68
Ferulic acid
F5H
5-Hydroxyferulic acid
2.1.1.68
Sinapic acid
2.4.1.120
1-O-Sinapoyl-β-D-glucose
2.3.1.92
Sinapoyl-malate
2.3.1.91
1.14.13.14
Ubiquonone biosynthesis
4-Hydroxystyrene
4.1.1.102
3.1.1.-
4.1.1.102
3,4-Dihydroxy-styrene
3.1.1.49
Sinapoyl-choline
6.2.1.12
6.2.1.12
6.2.1.12
6.2.1.12
6.2.1.12
6.2.1.12
2.3.1.133
1.14.13.36
2.3.1.133
p-Coumaroyl shikimic acid
Caffeoyl shikimic acid
trans-2-Hydroxy-cinnamate
Cinnamoyl-CoA
1.14.13.11
p-Coumaroyl-CoA
1.14.13.-
Caffeoyl-CoA
2.1.1.104
Feruloyl-CoA
5-Hydroxy-feruloyl-CoA
2.1.1.104
Sinapoyl-CoA
2.4.1.114
2.3.1.99
1.2.1.68
1.2.1.68
β-D-Glucosyl-2-coumarate
1.2.1.44
p-Coumaroyl quinic acid
2.3.1.133
1.14.13.36
2.3.1.133
1.2.1.44
1.2.1.44
1.2.1.44
1.2.1.44
Caffeoyl quinic acid
1.2.1.44
5.2.1.-
Cinnamaldehyde
p-Coumaraldehyde
1.14.13.-
Caffeyl-aldehyde
2.1.1.68
Coniferyl-aldehyde
F5H
5-Hydroxy-coniferaldehyde
2.1.1.68
Sinapaldehyde
β-D-Glucosyl-2-coumarinate
Flavonoid biosynthesis
1.1.1.195
1.1.1.195
1.1.1.195
1.1.1.195
1.1.1.195
Stilbenoid,diarylheptanoid and gingerol biosynthesis
3.2.1.21
p-Coumaryl alcohol
Caffeyl-alcohol
2.1.1.68
Coniferyl-alcohol
F5H
5-Hydroxy-coniferyl alcohol
2.1.1.68
Sinapyl alcohol
p-Coumaryl acetate
CFAT
Coniferyl acetate
CFAT
Coumarinate
3.2.1.126
3.2.1.126
3.2.1.126
1.1.1.318
1.1.1.319
1.11.1.7
1.11.1.21
1.1.1.318
1.1.1.319
1.11.1.7
1.11.1.21
1.11.1.7
1.11.1.21
1.11.1.7
1.11.1.21
2.4.1.111
2.4.1.111
2.4.1.111
Chavicol
Isochavicol
Eugenol
Isoeugenol
Coumarine
2.1.1.146
2.1.1.279
p-Hydroxy-phenyl lignin
4-Hydroxycinnamyl-alcohol 4-D-glucoside
2.1.1.146
2.1.1.146
2.1.1.279
Guaiacyl lignin
Coniferin
5-Hydroxy-guaiacyl lignin
Syringyl lignin
Syringin
Methylchavicol
Anethole
Methyleugenol
Isomethyleugenol
00940 4/5/16
(c) Kanehisa Laboratories

d

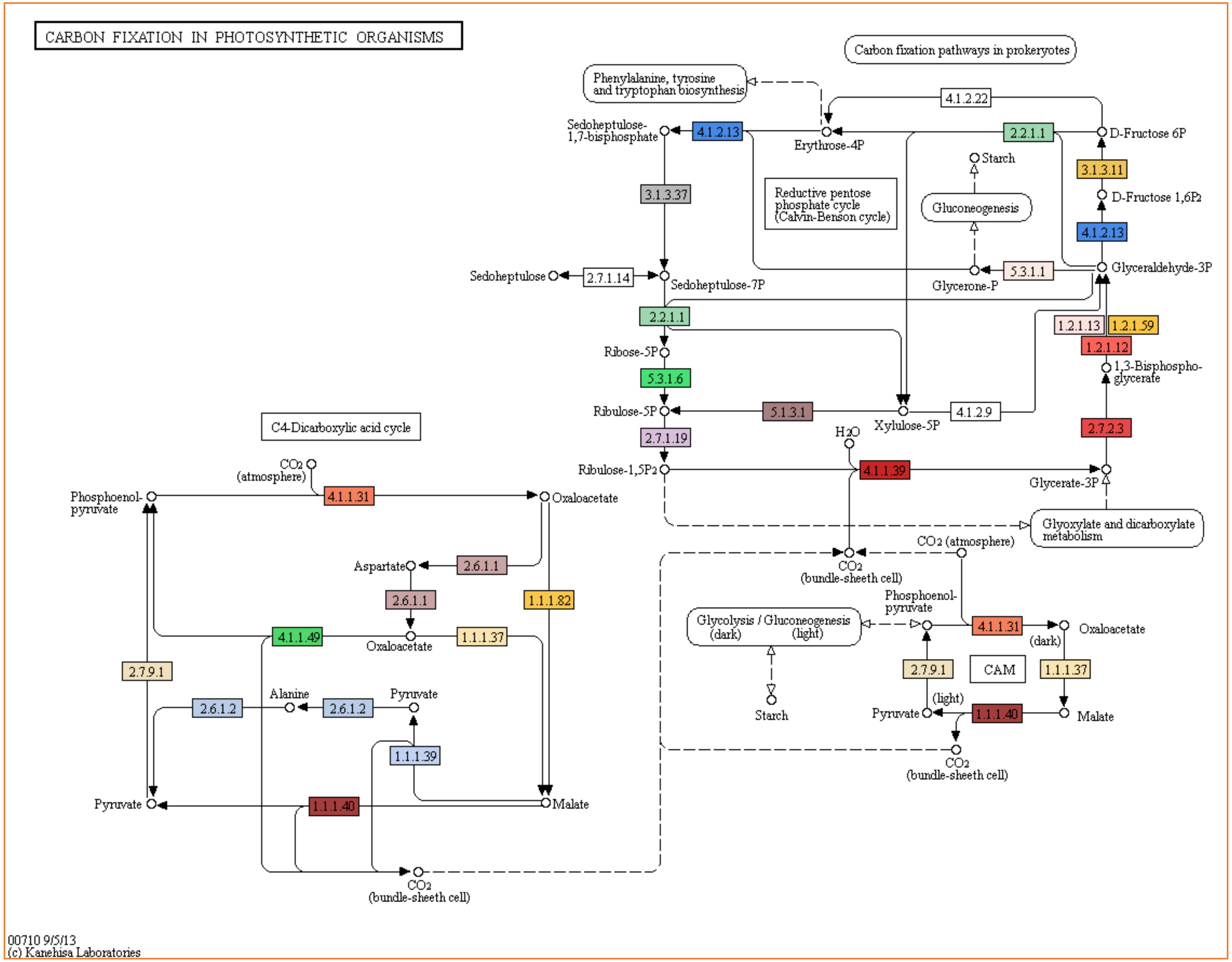

CARBON FIXATION IN PHOTOSYNTHETIC ORGANISMS
Carbon fixation pathways in prokaryotes
Phenylalanine, tyrosine and tryptophan biosynthesis
4.1.2.22
Sedoheptulose-1,7-bisphosphate
4.1.2.13
Erythrose-4P
2.2.1.1
D-Fructose 6P
Starch
3.1.3.11
3.1.3.37
Reductive pentose phosphate cycle (Calvin-Benson cycle)
Gluconeogenesis
D-Fructose 1,6P2
4.1.2.13
Glyceraldehyde-3P
5.3.1.1
Glycerone-P
Sedoheptulose
2.7.1.14
Sedoheptulose-7P
2.2.1.1
1.2.1.13
1.2.1.59
1.2.1.12
Ribose-5P
1,3-Bisphospho-glycerate
5.3.1.6
Ribulose-5P
5.1.3.1
Xylulose-5P
4.1.2.9
C4-Dicarboxylic acid cycle
2.7.1.19
H2O
2.7.2.3
Ribulose-1,5P2
4.1.1.39
Glycerate-3P
CO2 (atmosphere)
Phosphoenol-pyruvate
4.1.1.31
Oxaloacetate
Glyoxylate and dicarboxylate metabolism
CO2 (atmosphere)
CO2 (bundle-sheeth cell)
Aspartate
2.6.1.1
2.6.1.1
1.1.1.82
Phosphoenol-pyruvate
Glycolysis / Gluconeogenesis (dark) (light)
4.1.1.31
Oxaloacetate
(dark)
4.1.1.49
Oxaloacetate
1.1.1.37
2.7.9.1
2.7.9.1
CAM
1.1.1.37
(light)
Alanine
Pyruvate
2.6.1.2
2.6.1.2
Starch
Pyruvate
1.1.1.40
Malate
1.1.1.39
CO2 (bundle-sheeth cell)
Pyruvate
1.1.1.40
Malate
CO2 (bundle-sheeth cell)
00710 9/5/13
(c) Kanehisa Laboratories

Table S4. Sugarcane transcripts aligned against the sorghum genome

	**PacBio transcript isoforms**	***De novo* transcript contigs**
From total transcripts	77,164	397,230
% Transcript aligned to sorghum	69.44%	40.98%
From TransDecoder retained set	123,986	383,024
% Transcript aligned to sorghum	80.79%	70.05%
From Evigene predicted transcripts	40,783	32,023
% Transcript aligned to sorghum	78.68%	37.02%

Table S5. List of 164 selected genes from sugarcane and grass family used in the full-length assessment

Name / GI / Accession number	Length (bp)	Description
Sugarcane COMT	1479	Saccharum Caffeic acid 3-O-methyltransferase - Saccharum hybrid cultivar, complete
Sugarcane CWI	1734	Saccharum hybrid GT28 cell-wall invertase
Sugarcane SO_PAL_KC416026	2118	Saccharum hybrid cultivar CP69-1062 phenylalanine ammonia-lyase mRNA, complete cds.
HQ242713.1	1341	Saccharum hybrid cultivar glyceraldehyde-3-phosphate dehydrogenase gene, partial cds
gi|605371297|gb|KF732705.1|	1950	Saccharum hybrid cultivar Hsp70 (hsp70) mRNA, complete cds
SPSA	3906	Saccharum hybrid cultivar ROC22 sucrose phosphate synthase A (SPSA) mRNA, complete cds
Sugarcane UDPGP	1431	Saccharum hybrid cultivar SP80-3280 UDP-glucose-pyrophosphorylase (Ugpase-1) mRNA, complete cds
Sugarcane ndhD	1182	Saccharum NAD(P)H-quinone oxidoreductase chain H, complete
ANR02565.1	1665	Saccharum hybrid cultivar CP88-1762, 4-coumarate:coenzyme A ligase 1
AE009947.2	744	Saccharum hybrid cultivar SP-80-3280 NADH dehydrogenase subunit K
gi|47605312|gb|AY596588.1|	1185	Saccharum officinarum clone SCUTLR2030F03, complete sequence
Sugarcane SPS	3322	Saccharum officinarum mRNA for Sucrose-Phosphate Synthase, partial cds
Sugarcane SUSYII	2409	Saccharum officinarum sucrose synthase (Susy2)
Sugarcane UDPGP-1	793	Saccharum officinarum UDP-glucose pyrophosphorylase gene, partial cds
Sugarcane SPP	1520	Saccharum Phosphate phosphatase S. off mRNA, complete cds
gi|47605262|gb|AY596538.1|	1227	Saccharum officinarum similar to hypothetical protein LOC100217086 [Zea mays]

Sugarcane SUT1-H1	1566	Saccharum spontaneum SUT1-h1 gene, complete cds
Sugarcane SUT1-H2	1566	Saccharum spontaneum SUT1-h2 gene, complete cds
Sugarcane SUT4-H1	1506	Saccharum spontaneum SUT4-h1 gene, complete cds
Sugarcane SUT6-H3	1605	Saccharum spontaneum SUT6-h3 gene, complete cds
Sugarcane UGD gene	2024	Saccharum UDG isoform
gi|955716488|ref|XM_012844651.2|	1826	Setaria italica beta-glucosidase 22-like (LOC101765240), mRNA
gi|955724816|ref|XM_004983344.3|	6247	Setaria italica callose synthase 10 (LOC101773496), mRNA
gi|955718913|ref|XM_014805168.1|	1469	Setaria italica E3 ubiquitin-protein ligase At4g11680-like (LOC101765163), mRNA
gi|955718355|ref|XM_014805131.1|	1910	Setaria italica WAT1-related protein At3g45870 (LOC101767993), transcript variant X2, mRNA
gi|20530128|dbj|AB084897.1|	1862	Sorghum bicolor ALDH2a mRNA for mitochondrial aldehyde dehydrogenase, complete cds
gi|242032582|ref|XM_002463641.1|	1519	Sorghum bicolor hypothetical protein, mRNA
gi|242032910|ref|XM_002463805.1|	1822	Sorghum bicolor hypothetical protein, mRNA
gi|242033194|ref|XM_002463947.1|	3405	Sorghum bicolor hypothetical protein, mRNA
gi|242033196|ref|XM_002463948.1|	4119	Sorghum bicolor hypothetical protein, mRNA
gi|242033964|ref|XM_002464332.1|	1792	Sorghum bicolor hypothetical protein, mRNA
gi|242034958|ref|XM_002464829.1|	1495	Sorghum bicolor hypothetical protein, mRNA
gi|242035106|ref|XM_002464903.1|	4512	Sorghum bicolor hypothetical protein, mRNA
gi|242035394|ref|XM_002465047.1|	1544	Sorghum bicolor hypothetical protein, mRNA
gi|242035622|ref|XM_002465161.1|	1951	Sorghum bicolor hypothetical protein, mRNA
gi|242036178|ref|XM_002465439.1|	1670	Sorghum bicolor hypothetical protein, mRNA
gi|242037182|ref|XM_002465941.1|	801	Sorghum bicolor hypothetical protein, mRNA
gi|242037302|ref|XM_002466001.1|	3242	Sorghum bicolor hypothetical protein, mRNA
gi|242038476|ref|XM_002466588.1|	1838	Sorghum bicolor hypothetical protein, mRNA
gi|242038522|ref|XM_002466611.1|	988	Sorghum bicolor hypothetical protein, mRNA
gi|242039430|ref|XM_002467065.1|	1006	Sorghum bicolor hypothetical protein, mRNA
gi|242039564|ref|XM_002467132.1|	1482	Sorghum bicolor hypothetical protein, mRNA
gi|242041386|ref|XM_002468043.1|	2527	Sorghum bicolor hypothetical protein, mRNA
gi|242041786|ref|XM_002468243.1|	2198	Sorghum bicolor hypothetical protein, mRNA
gi|242044161|ref|XM_002459907.1|	1182	Sorghum bicolor hypothetical protein, mRNA
gi|242046047|ref|XM_002460850.1|	2447	Sorghum bicolor hypothetical protein, mRNA
gi|242049217|ref|XM_002462308.1|	1789	Sorghum bicolor hypothetical protein, mRNA
gi|242049363|ref|XM_002462381.1|	1008	Sorghum bicolor hypothetical protein, mRNA
gi|242050543|ref|XM_002462971.1|	1265	Sorghum bicolor hypothetical protein, mRNA
gi|242051095|ref|XM_002463247.1|	1182	Sorghum bicolor hypothetical protein, mRNA
gi|242051448|ref|XM_002454825.1|	798	Sorghum bicolor hypothetical protein, mRNA
gi|242051670|ref|XM_002454936.1|	1618	Sorghum bicolor hypothetical protein, mRNA
gi|242052306|ref|XM_002455254.1|	1975	Sorghum bicolor hypothetical protein, mRNA
gi|242053618|ref|XM_002455910.1|	1304	Sorghum bicolor hypothetical protein, mRNA
gi|242053696|ref|XM_002455949.1|	1848	Sorghum bicolor hypothetical protein, mRNA

gi|242055374|ref|XM_002456788.1|	1845	Sorghum bicolor hypothetical protein, mRNA
gi|242055932|ref|XM_002457067.1|	4769	Sorghum bicolor hypothetical protein, mRNA
gi|242055986|ref|XM_002457094.1|	1799	Sorghum bicolor hypothetical protein, mRNA
gi|242058890|ref|XM_002458546.1|	4025	Sorghum bicolor hypothetical protein, mRNA
gi|242060743|ref|XM_002451616.1|	2089	Sorghum bicolor hypothetical protein, mRNA
gi|242060917|ref|XM_002451703.1|	1571	Sorghum bicolor hypothetical protein, mRNA
gi|242062813|ref|XM_002452651.1|	1363	Sorghum bicolor hypothetical protein, mRNA
gi|242063269|ref|XM_002452879.1|	1965	Sorghum bicolor hypothetical protein, mRNA
gi|242063281|ref|XM_002452885.1|	2385	Sorghum bicolor hypothetical protein, mRNA
gi|242064485|ref|XM_002453487.1|	2806	Sorghum bicolor hypothetical protein, mRNA
gi|242066325|ref|XM_002454407.1|	773	Sorghum bicolor hypothetical protein, mRNA
gi|242066553|ref|XM_002454521.1|	1346	Sorghum bicolor hypothetical protein, mRNA
gi|242066575|ref|XM_002454532.1|	1078	Sorghum bicolor hypothetical protein, mRNA
gi|242066625|ref|XM_002454557.1|	2111	Sorghum bicolor hypothetical protein, mRNA
gi|242066927|ref|XM_002454708.1|	1119	Sorghum bicolor hypothetical protein, mRNA
gi|242066983|ref|XM_002454736.1|	1631	Sorghum bicolor hypothetical protein, mRNA
gi|242067418|ref|XM_002448941.1|	1455	Sorghum bicolor hypothetical protein, mRNA
gi|242068230|ref|XM_002449347.1|	1705	Sorghum bicolor hypothetical protein, mRNA
gi|242068758|ref|XM_002449611.1|	1597	Sorghum bicolor hypothetical protein, mRNA
gi|242071698|ref|XM_002451081.1|	1041	Sorghum bicolor hypothetical protein, mRNA
gi|242071754|ref|XM_002451109.1|	1745	Sorghum bicolor hypothetical protein, mRNA
gi|242072767|ref|XM_002446275.1|	1177	Sorghum bicolor hypothetical protein, mRNA
gi|242075233|ref|XM_002447508.1|	5674	Sorghum bicolor hypothetical protein, mRNA
gi|242076571|ref|XM_002448177.1|	1308	Sorghum bicolor hypothetical protein, mRNA
gi|242076721|ref|XM_002448252.1|	2063	Sorghum bicolor hypothetical protein, mRNA
gi|242077039|ref|XM_002448411.1|	1179	Sorghum bicolor hypothetical protein, mRNA
gi|242077888|ref|XM_002443668.1|	2727	Sorghum bicolor hypothetical protein, mRNA
gi|242079252|ref|XM_002444350.1|	1778	Sorghum bicolor hypothetical protein, mRNA
gi|242080040|ref|XM_002444744.1|	1658	Sorghum bicolor hypothetical protein, mRNA
gi|242080108|ref|XM_002444778.1|	1188	Sorghum bicolor hypothetical protein, mRNA
gi|242080214|ref|XM_002444831.1|	1613	Sorghum bicolor hypothetical protein, mRNA
gi|242081000|ref|XM_002445224.1|	1227	Sorghum bicolor hypothetical protein, mRNA
gi|242082014|ref|XM_002445731.1|	1534	Sorghum bicolor hypothetical protein, mRNA
gi|242082080|ref|XM_002445764.1|	2193	Sorghum bicolor hypothetical protein, mRNA
gi|242083377|ref|XM_002442069.1|	2203	Sorghum bicolor hypothetical protein, mRNA
gi|242083883|ref|XM_002442322.1|	1200	Sorghum bicolor hypothetical protein, mRNA
gi|242085861|ref|XM_002443311.1|	522	Sorghum bicolor hypothetical protein, mRNA
gi|242086207|ref|XM_002443484.1|	1492	Sorghum bicolor hypothetical protein, mRNA
gi|242086654|ref|XM_002439115.1|	787	Sorghum bicolor hypothetical protein, mRNA

gi|242086658|ref|XM_002439117.1|	1811	Sorghum bicolor hypothetical protein, mRNA
gi|242087790|ref|XM_002439683.1|	2015	Sorghum bicolor hypothetical protein, mRNA
gi|242088350|ref|XM_002439963.1|	1507	Sorghum bicolor hypothetical protein, mRNA
gi|242089614|ref|XM_002440595.1|	2063	Sorghum bicolor hypothetical protein, mRNA
gi|242090186|ref|XM_002440881.1|	1143	Sorghum bicolor hypothetical protein, mRNA
gi|242090710|ref|XM_002441143.1|	2370	Sorghum bicolor hypothetical protein, mRNA
gi|242091316|ref|XM_002441446.1|	2309	Sorghum bicolor hypothetical protein, mRNA
gi|242091426|ref|XM_002441501.1|	1762	Sorghum bicolor hypothetical protein, mRNA
gi|242093697|ref|XM_002437294.1|	1012	Sorghum bicolor hypothetical protein, mRNA
gi|242093983|ref|XM_002437437.1|	1610	Sorghum bicolor hypothetical protein, mRNA
gi|242095229|ref|XM_002438060.1|	1500	Sorghum bicolor hypothetical protein, mRNA
gi|242095355|ref|XM_002438123.1|	3626	Sorghum bicolor hypothetical protein, mRNA
gi|242095745|ref|XM_002438318.1|	2432	Sorghum bicolor hypothetical protein, mRNA
gi|242096971|ref|XM_002438931.1|	1666	Sorghum bicolor hypothetical protein, mRNA
gi|257220645|gb|GQ380441.1|	2114	Sorghum bicolor S-adenosylmethionine decarboxylase 1 (SAMDC1) mRNA, complete cds
Phytochrome_B	1128	Tripsacum dactyloides (Gama grass), Phytochrome B - complete
ZADH	1140	Zea mays alcohol dehydrogenase 1 (adh1) gene, adh1-F allele, complete cds
gi|1002244122|ref|XM_015770788.1|	1267	Oryza sativa Japonica Group tuliposide A-converting enzyme 2, chloroplastic (LOC4329717), mRNA
gi|670408373|ref|XM_008647329.1|	4418	Zea mays BAG family molecular chaperone regulator 6 (LOC103626967), transcript variant X2, mRNA
ZMces7	3968	Zea mays cellulose synthase 7 (cesa7), mRNA
gi|1013823631|ref|NM_001137144.2|	1215	Zea mays chlorophyll a-b binding protein (LOC100191715), mRNA
ZAQP	753	Zea mays clone 1290530 aquaporin TIP1.1 mRNA, complete cds
gi|195623377|gb|EU961401.1|	758	Zea mays clone 235148 hypothetical protein mRNA, complete cds
gi|195625117|gb|EU962271.1|	1733	Zea mays clone 241424 hypothetical protein mRNA, complete cds
gi|195628723|gb|EU964074.1|	1474	Zea mays clone 275729 GSDL-motif lipase mRNA, complete cds
gi|195638487|gb|EU966594.1|	1166	Zea mays clone 295518 mRNA sequence
gi|195642391|gb|EU968546.1|	1857	Zea mays clone 322105 mRNA sequence
gi|195649936|gb|EU972318.1|	1310	Zea mays clone 379594 mRNA sequence
gi|195653468|gb|EU974084.1|	1613	Zea mays clone 439404 mRNA sequence
gi|212720764|ref|NM_001139201.1|	1354	Zea mays cytochrome c1, heme protein (LOC100194151), mRNA
gi|670439658|ref|XM_008661964.1|	6005	Zea mays dedicator of cytokinesis protein 7 (LOC103639174), transcript variant X3, mRNA
gi|238015231|gb|BT088298.1|	824	Zea mays full-length cDNA clone ZM_BFc0123L11 mRNA, complete cds
gi|670429787|ref|XM_008657493.1|	4152	Zea mays gigantea like1a (gigz1a), transcript variant X2, mRNA
gi|226528388|ref|NM_001156739.1|	1559	Zea mays GTP-binding protein PTD004 (LOC100283841), mRNA
Zea mays hexokinase 2 ZHK	1524	Zea mays hexokinase 2 (hex2), mRNA
gi|670357938|ref|XM_008675671.1|	2222	Zea mays hypothetical protein (LOC100191941), transcript variant X1, mRNA
gi|670368715|ref|XM_008667350.1|	4515	Zea mays indole-3-acetaldehyde oxidase-like (LOC103644157), mRNA
gi|670412398|ref|XM_008649133.1|	1409	Zea mays LOC100192895 (TIDP2649), transcript variant X1, mRNA
gi|670358412|ref|XM_008666384.1|	1188	Zea mays LOC100194394 (pco132275a), transcript variant X4, mRNA

gi|670428088|ref|XM_008656584.1|	2840	Zea mays LOC100279465 (IDP77), transcript variant X1, mRNA
gi|670388190|ref|XM_008676372.1|	1710	Zea mays mitochondrial thiamine pyrophosphate carrier 1-like (LOC103650800), transcript variant X1, mRNA
gi|226506199|ref|NM_001156163.1|	1689	Zea mays MRS2-10 (LOC100283261), mRNA
gi|21207784|gb|AY104706.1|	1382	Zea mays PCO076122 mRNA sequence
gi|187950314|gb|AY103975.2|	1833	Zea mays PCO080341 mRNA sequence
gi|21211330|gb|AY108252.1|	1456	Zea mays PCO090236 mRNA sequence
gi|670411327|ref|XM_008648571.1|	2709	Zea mays premnaspirodiene oxygenase-like (LOC103628355), mRNA
gi|670375973|ref|XM_008671011.1|	4551	Zea mays probable serine/threonine-protein kinase GCN2 (LOC103646282), transcript variant X2, mRNA
gi|226506901|ref|NM_001154635.1|	2255	Zea mays protein kinase (LOC100281715), mRNA
gi|293333669|ref|NM_001175179.1|	1664	Zea mays putative dnaJ chaperone family protein (LOC100382437), mRNA
gi|670398460|ref|XM_008681049.1|	6129	Zea mays separase (LOC103654220), transcript variant X3, mRNA
gi|819231710|ref|NM_001308514.1|	1913	Zea mays serine hydroxymethyltransferase (IDP527), mRNA
gi|212275727|ref|NM_001137354.1|	2893	Zea mays uncharacterized LOC100191930 (LOC100191930), mRNA
gi|670357954|ref|XM_008677736.1|	4825	Zea mays uncharacterized LOC100192064 (LOC100192064), transcript variant X1, mRNA
gi|670371790|ref|XM_008668960.1|	5668	Zea mays uncharacterized LOC100193501 (LOC100193501), transcript variant X2, mRNA
gi|212722793|ref|NM_001139095.1|	1311	Zea mays uncharacterized LOC100194034 (LOC100194034), mRNA
gi|670420277|ref|XM_008652741.1|	4580	Zea mays uncharacterized LOC100216554 (LOC100216554), transcript variant X1, mRNA
gi|226491258|ref|NM_001149150.1|	2488	Zea mays uncharacterized LOC100274892 (pco094349), mRNA
gi|910242272|ref|NM_001152011.2|	1432	Zea mays uncharacterized LOC100278877 (LOC100278877), mRNA
gi|670421027|ref|XR_555418.1|	4694	Zea mays uncharacterized LOC100280438 (LOC100280438), transcript variant X2, misc_RNA
gi|226504287|ref|NM_001155050.1|	1606	Zea mays uncharacterized LOC100282138 (pco117051), mRNA
gi|226533416|ref|NM_001158924.1|	2503	Zea mays uncharacterized LOC100286036 (AY104511), mRNA
gi|670373881|ref|XM_008670073.1|	2317	Zea mays uncharacterized LOC100381853 (LOC100381853), transcript variant X3, mRNA
gi|293336488|ref|NM_001176146.1|	1990	Zea mays uncharacterized LOC100383498 (csu43), mRNA
gi|293332010|ref|NM_001177046.1|	1652	Zea mays uncharacterized LOC100384528 (LOC100384528), mRNA
gi|670367162|ref|XM_008666731.1|	1474	Zea mays uncharacterized LOC103643562 (LOC103643562), mRNA
gi|670368670|ref|XM_008667333.1|	3289	Zea mays uncharacterized LOC103644136 (LOC103644136), transcript variant X2, mRNA
gi|670391932|ref|XM_008678105.1|	6091	Zea mays uncharacterized LOC103652548 (LOC103652548), mRNA
gi|670398368|ref|XM_008681016.1|	4750	Zea mays uncharacterized LOC103654189 (LOC103654189), transcript variant X2, mRNA
gi|670445886|ref|XR_560425.1|	1871	Zea mays WAT1-related protein At5g64700-like (LOC103641647), transcript variant X2, misc_RNA
ZMCeS6	3240	Zea mays ZM PATENT Sequence 5 from Patent WO0179516
