# Supplementary material for: A survey of the complex transcriptome from the highly polyploid sugarcane genome using full-length isoform sequencing and de novo assembly from short read sequencing
Source: BMC Genomics. 2017 May 22;18:395. doi: 10.1186/s12864-017-3757-8 (PMC5440902; doi:10.1186/s12864-017-3757-8)
Supplement: Supplementary file 2 — Additional Method: More details of PacBio Iso-Seq library preparation and data processing. Figure S7 Non-normalized and normalized cDNA libraries used in this study. a, RNA profiles of individual sample pooling and all sample pooling in Bioanalyser. b, Two non-normalized samples on agarose 1.2%, mouse RNA was used as control. c, Results of normalized cDNA samples treated with 1U and 0.5U DSN. d, A comparison between the non-normalized and normalized cDNA profiles. Figure S8 PacBio Iso-Seq data processing and read correction (adopted from Pacific Biosciences). (PDF 669 kb) [file 12864_2017_3757_MOESM2_ESM.pdf]

Additional Method: PacBio Iso-Seq library preparation and data processing

Non-normalized cDNA library preparation

The non-normalized library was followed the SMARTer PCR cDNA synthesis kit (ClonTech, Takara Bio Inc., Shiga, Japan) and KAPA HiFi PCR kit (Kapa Biosystems, Boston, USA); while the normalized library was generated from an aliquot of non-normalized cDNA using the Trimmer-2 kit (Evrogen, Moscow, Russia). Approximately 1 µg of total RNA of pooled sample and 1 µg a mouse liver total RNA sample as a positive control, were used in a single step of cDNA first stranded synthesis. The first strand cDNA synthesis reaction was catalyzed by SMARTScribe Reverse Transcriptase, using a 3' SMART CDS Primer IIA (5'–AAGCAGTGGTATCAACGCAGAGTACT$_{(30)}$N$_{-1}$N–3') and a template-switching mechanism of same enzyme using the SMARTer II A Oligonucleotide (5'–AAGCAGTGGTATCAACGCAGAGTACXXXXX–3'), to generate full-length single stranded cDNA containing the complete 5' end of the mRNA molecules. The PCR reaction was carried out in a hot-lid a Bio-Rad T100^{TM} Thermal Cycler (Bio-Rad Thermal Cycler, Bio-Rad, Hercules, CA, USA), with following conditions: annealing at 70°C for 3 min, slow ramp to 42°C for 2 min (to allow 3' SMART CDS Primer IIA binding), first-strand synthesis at 42°C for 90 min and terminating of reaction at 70°C for 10 min. The first-strand reaction product (10 µl) was then diluted five times with 40 µl elution buffer containing Tris-HCl 10mM (pH 8.0), resulting in final volume of 50 µl of first-strand cDNA. For PCR optimization, we combined in a total reaction volume of 50 µl; 10 µl diluted first-strand cDNA, 3.2 µl of 5' PCR Primer IIA (5'-AAGCAGTGGTATCAACGCAGA GTAC-3') (batch number: SD00325122, CloneTech); and KAPA PCR kit reagents (10 µl KAPA HiFi Fidelity 5X buffer, 1.5 µl KAPA dNTP mix 10 mM, and 1U of KAPA HiFi enzyme). The PCR cycle optimization was performed with the following conditions: initial denaturation at 95°C for 2 min, followed by 10 cycles of 98°C for 20s, 65°C for 15s and 72°C for 4 min, lid temperature of 105°C. After the 11th cycle of PCR reaction, 5 µl of the reaction was removed, and subsequently, after every two additional cycles of the reaction, 5 µl was removed at after every three cycles from the 13th to 31st cycles. The PCR products were assessed by resolving in 1.5% agarose (w/v) containing SYBR® Safe DNA Gel Stain (ThermoFisher Scientific) prepared in 0.5X TBE buffer, visualized under a GelDoc system (Uvitec Ltd., Cambridge, UK) to determine the optimal cycles. A 1kb plus DNA ladder (ThermoFisher) was used to estimate the size of the PCR products. The optimal cycle number then was used for a large-scale PCR reaction to amplify double stranded cDNA with the same settings as the optimization step, with initial denaturation (95°C/2min), followed by 18 cycles (98°C/20s, 65°C/15s and 72°C/4 min), and a final extension step (72°C/5 min). The PCR amplified products were resolved in 1.5% agarose, and by using Qubit dsDNA high sensitivity assay on a Qubit Fluorometer 3.0 (ThermoFirsher Scientific) before subjected to Bioanalyser and the DNA chip using the DNA 12000 Kit reagents and assay (Agilent technologies, Santa Clara, CA, USA) for the final estimation of the DNA yield.

Normalized cDNA library preparation

The amplified cDNA was normalized by Trimmer-2 kit, to decrease the prevalence of high abundant transcripts and to capture the rare transcripts. This normalization kit relies on the nucleic acid hybridization

(Zhulidov et al. 2005) and unique properties of duplex-specific nuclease (DSN) isolated from Kamchatka crab which preferentially cleaves double stranded (ds) DNA compared to single stranded DNA and RNA (Shagin et al. 2002). This results in a reduction in the abundant transcripts and normalization of the transcript copies in the total cDNA library. The amplified non-normalized cDNA from previous step was purified using the QIAquick PCR purification kit (Qiagen), and 1 µg of purified cDNA was precipitated using 0.1 volume of 3M sodium acetate (pH 4.8) and 2.5 volumes of 98% (v/v) ethanol. The cDNA pellet was centrifuged at maximum speed for 15 min, washed with 100 µl of 80% ethanol twice. The purified and precipitated cDNA was re-suspended in RNase-free water at the final concentration of 100 ng/µl. For each cDNA sample, 10 µl ds cDNA was combined with 4 µl of hybridization buffer and 2 µl of sterile RNase-free water, resulting in a final solution of 16 µl. The final solution was aliquoted into four tubes (4 µl each), incubated at 98°C for 2 min and 68°C for 5 h using a thermal cycler. The four hybridized cDNA tubes were subjected to DSN treatments, by adding 5 µl DSN master buffer followed by incubating at 68°C for 10 min, and subsequently adding 1 µl DSN solution containing 1U DSN, 0.5U DSN, 0.25U DSN and 0U DSN (control). The tube temperature was controlled at 68°C by keeping in the thermal cycler to avoid non-specific digestion leading to the decreasing the normalization efficiency. The DSN treatment was carried out at 68°C for 25 min, then 5 µl of DSN stop solution was added to each tube, mixed and kept at 68°C for further 5 min to terminate the digestion reaction. Tubes were placed on ice and 25 µl of sterile RNase-free water was added to each tube. The treated cDNA tubes were used for cDNA amplification to recover the normalized cDNA. Every 1 µl of treated cDNA solution was combined with 3.2 µl of 5' PCR Primer IIA; 10 µl KAPA HiFi Fidelity 5X buffer; 1.5 µl KAPA dNTP mix 10 mM; 1U of KAPA HiFi enzyme; and 33.3 µl RNase-free water, resulting in a total reaction solution of 50 µl. The PCR cycle optimization was performed with the following conditions: initial denaturation at 95°C for 2 min, followed by 7 cycles of 98°C (20s), 65°C (15s) and 72°C (4 min), using a heated lid of 105°C. After the 7th cycle of PCR reaction, three tubes treated with DSN (also referred to as experimental tubes) were put on ice and the control tube was used for PCR cycle optimization. An aliquot of 5 µl from the seven-cycled control tube was removed, and the remaining solution was run for two-additional-cycle steps (for a total of nine, 11 and 13 cycles), and 5 µl was removed at each step. The PCR products were assessed by resolving in 1.5% agarose, and used to determine the optimal cycles for control sample. The total cycles for PCR amplification of normalized cDNA tubes were determined according to the manufacturer, to be optimal cycles of the control tube (9 cycles), plus additional nine cycles (for a total of 18). Similarly, the results from each of these steps were analyzed on 1.5% agarose gel, and the final cycles determined to be 18 for all three treated samples. Finally, 10 large-scale PCR reactions (volume of 50 µl per reaction) were carried out for each of the treated samples to recover enough cDNA for sequencing. The PCR conditions were as same as for the optimization of the control tube, with a final extension step (72°C/5 min). The quality and yield of the final PCR amplified products were analyzed in 1.5% agarose, Qubit dsDNA assay and Bioanalyser DNA chip, as described earlier for non-normalized samples.

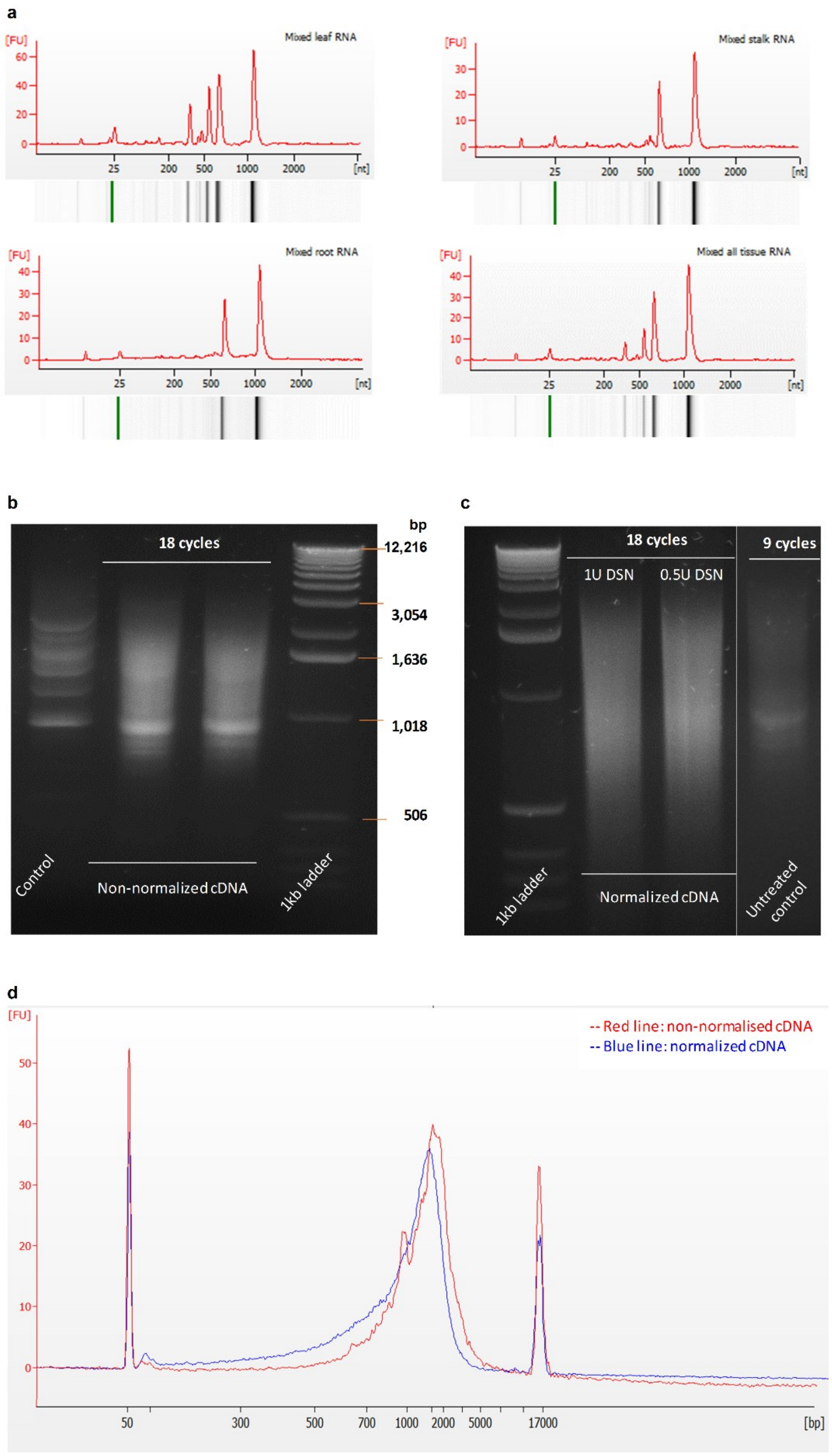

Figure S7. Non-normalized and normalized cDNA libraries used in this study. a, RNA profiles of individual sample pooling and all sample pooling in Bioanalyser. **b**, Two non-normalized samples on agarose 1.2%, mouse RNA was used as control. **c**, Results of normalized cDNA samples treated with 1U and 0.5U DSN. **d**, A comparison between the non-normalized and normalized cDNA profiles.

Size fraction of PacBio Iso-Seq libraries

The large-scale PCR products from 1U DSN treated sample were purified using the 1X volume of AMPure® PB magnetic beads (Beckman-Coulter, Brea, CA, USA), washed with 70% ethanol and then eluted in elution buffer. The final concentration was determined by using Qubit system. To eliminate the length bias during library sequencing towards the short transcripts (1 kb to 1.5 kb) in the no-size selection procedure, the cDNA library was size-fragmented according to the PacBio Iso-Seq protocol, employing the BluePinpin system (Sage Science). Four bins (0.5-2.5 kb, 2-3.5 kb, 3-6 kb, and 5-10 kb) of the non-normalized library and two bins (0.5-2.5 kb and 2-3.5 kb) of the normalized cDNA library were amplified separately to recover enough cDNA for PacBio sequencing (8 µg each bin required). The same KAPA PCR kit reagents, SMARTer 5'PCR Primer IIA and PCR conditions as previous experiments were applied using the optimal cycles for each bin, 0.5-2.5 kb (8-12 cycles/1 min), 2-3.5 kb (10-12 cycles/1 min 45 sec), 3-6 kb (12-15 cycles/3 min), and 5-10 kb (15 cycles/4 min 30 sec). The amplified products of each bin were pooled together into 1.5 ml tubes, purified with 1X AMPure PB beads and eluted in elution buffer. The amplified cDNA then was subjected to DNA damage and ends repair, adaptor-ligation for Iso-Seq cDNA libraries and sequenced in six SMRT cells (Pacific Biosciences) for each corresponding bins. Purification, size selection, PCR amplification and sequencing of all cDNA bins were conducted on a PacBio RS II instrument, at the Ramaciotti Centre for Genomics, The University of New South Wales, Australia.

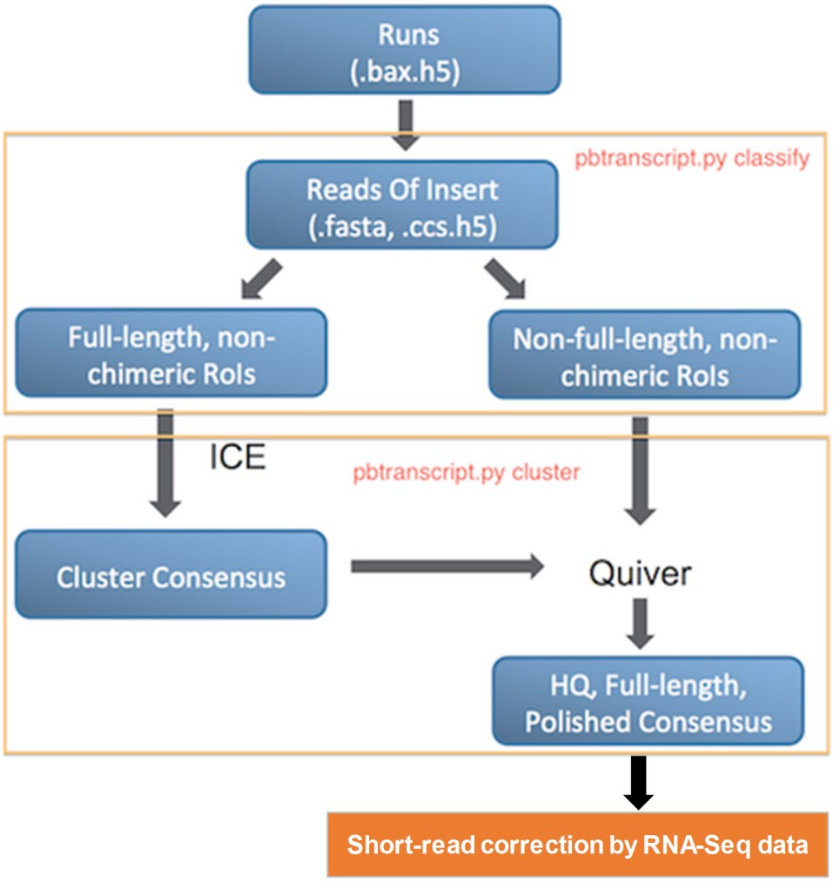

Figure S8. PacBio Iso-Seq data processing and read correction (adopted from Pacific Biosciences)
